# Insights Into the Local Heating Effects in Triple‐Cation Mixed‐Halide Perovskite Cells: Charge Dynamics, Coherent Phonons, Anion Segregation

**DOI:** 10.1002/smll.202408541

**Published:** 2025-01-16

**Authors:** Jacek J. Baranowski, Sanjay Sahare, Mykhailo Solovan, Patryk Florczak, Marcin Ziółek

**Affiliations:** ^1^ Faculty of Physics and Astronomy Adam Mickiewicz University Poznan 61–614 Poland; ^2^ NanoBioMedical Centre Adam Mickiewicz University Poznan 61–614 Poland

**Keywords:** anion segregation, coherent longitudinal acoustic phonons (CLAP), femtosecond transient absorption (TA), local heating, perovskite solar cells (PSCs), triple‐cation perovskites

## Abstract

The behavior of triple‐cation mixed halide perovskite solar cells (PSCs) under ultrashort laser pulse irradiation at varying fluences is investigated, with a focus on local heating effects observed in femtosecond transient absorption (TA) studies. The carrier cooling time constant is found to increase from 230 fs at 2 µJ cm⁻^2^ to 1.3 ps at 2 mJ cm⁻^2^ while the charge population decay accelerates from tens of nanoseconds to the picosecond range within the same fluence range. At fluences between 0.5 and 5 mJ cm^2^, distinct oscillations in the TA signal (≈1.1 MHz) reveal the presence of coherent longitudinal acoustic phonons (CLAP). These phonons induce lattice strain propagating at the speed of sound through the perovskite layer and exhibit relatively long damping times. TA spectra further reveal a partially reversible segregation of iodide and bromide anions under pulsed excitation. Interestingly, higher local heating at increased pump fluences slows the segregation process, with time constants extending from 40 min at low fluences to 110 min at high fluences. However, the continuous irradiation results in significantly smaller segregation effects compared to ultrashort pulse irradiation.

## Graphical abstract

Triple cation mixed halide perovskite solar cells are investigated upon irradiation with ultrashort laser pulses causing different local heating conditions. Fluence‐dependent carrier cooling and charge population decay rate constants are revealed. The oscillations of transient absorption (TA) signal due to coherent longitudinal acoustic phonons (CLAP) are observed. Anion segregation is observed and its time constants increase with higher pulse fluence.

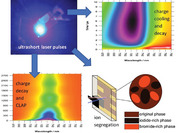

## 1 Introduction

Perovskites drew major attention in photovoltaics in 2009 when T. Miyasaka et al. developed the first hybrid organometal halide perovskite solar cell (PSC) with a 3.8% energy conversion efficiency.^[^ ^1^ ^]^ Since then, perovskite‐based photovoltaics (PVs) have become a focal point of research, with the efficiency of single‐junction solar cells reaching a record‐breaking 26.7% to date.^[^ ^2^ ^]^ Numerous perovskite compositions have been explored to optimize solar cell performance using material engineering.^[^ ^3^ ^]^ By mixing bromide, chloride, and iodide anions in various proportions to create mixed‐halide perovskites, researchers can fine‐tune the material's bandgap.^[^ ^4^ ^]^ Additionally, incorporating new cations, such as formamidinium (FA) or caesium, has proven effective in enhancing the stability of perovskite‐based solar cells. Apart from the advancements in device efficiency and structural investigations, significant attention has been devoted to study the photophysical processes occurring in metal halide perovskites following photoexcitation.^[^ ^5^, ^6^, ^7^, ^8^ ^]^ These studies offer valuable insights into the fundamental properties of perovskites, enabling researchers to refine material compositions, improve device architectures, and overcome critical challenges such as efficiency limitations, stability issues, and scalability in optoelectronic devices, including PVs, light‐emitting diodes (LEDs), and photodetectors.

Transient absorption (TA) spectroscopy is a powerful technique for exploring ultrafast photophysical processes in PSCs. It enables researchers to unravel complex interactions among charge carriers, excitons, and the perovskite lattice.^[^ ^8^, ^9^, ^10^, ^11^ ^]^ Specifically, it allows to study ultrafast phenomena such as hot carrier thermalization, nonlinear recombination, and interactions like electron‐electron and electron‐phonon coupling, which significantly influence the device performance and long‐term stability.^[^ ^6^, ^9^, ^12^, ^13^ ^]^ Further, it can investigate decay dynamics of photoexcited electron and hole populations within the perovskite's conduction and valence bands, which reveal the recombination pathways including first‐order trap‐assisted processes, as well as second‐order radiative and third‐order Auger recombination.^[^ ^6^, ^7^, ^8^ ^]^ The decay is also influenced by the charge transport layers, which accelerate recombination rates;^[^ ^6^, ^9^ ^]^ therefore, it is essential to investigate such effects in the complete device. Moreover, TA spectroscopy offers insights into how the initial spatial distribution of charges evolves over time through diffusion processes, which occur on time scales ranging from hundreds of picoseconds to several nanoseconds.^[^ ^6^, ^9^, ^14^ ^]^

The commercialization of perovskite technology is still hindered by stability issues, primarily due to its sensitivity to degradation when exposed to water or oxygen.^[^ ^15^ ^]^ In addition, ion migration across the crystal lattice, driven by vacancies, leads to the formation of ion barriers at the interfaces, which in turn reduces the PVs performance. A related phenomenon in mixed‐halide perovskites is anion segregation, first described by Hoke et al. in MAPb(Br_x_I_1‐x_)_3_,^[^ ^16^ ^]^ manifesting as a significant shift in the photoluminescence spectrum caused by the formation of bromide‐rich high‐bandgap domains and iodide‐rich low‐bandgap domains upon illuminations, which act as recombination centers and ultimately limit solar cell efficiency and long‐term stability.^[^ ^17^, ^18^, ^19^ ^]^ A couple of theories and models have already been developed to explain the mechanism of phase segregation.^[^ ^19^, ^20^, ^21^, ^22^ ^]^ The most important approaches are: (i) thermodynamic origin, where the band offset between the segregated domains results in a positive free energy, promoting phase separation; (ii) a polaron‐induced lattice strain gradient, which facilitates the nucleation of low‐bandgap domains; and (iii) the presence of a local electric field, which creates a charge carrier gradient, driving segregation. The origin of ion segregation can be attributed to trapped charge carriers^[^ ^23^ ^]^ or charge accumulation on surfaces,^[^ ^24^ ^]^ supporting the third model. Still, many important aspects of this effect are yet to be understood fully.^[^ ^25^ ^]^

The effect of light intensity on ion segregation remains unclear, with conflicting findings reported. Some studies suggest that ion segregation increases with higher irradiance, albeit not in a linear fashion,^[^ ^23^, ^26^ ^]^ while others describe the opposite behavior.^[^ ^27^ ^]^ Similarly, recent reports on the temperature influence show conflicting results, possibly due to variations in the perovskite compositions studied.^[^ ^28^, ^29^ ^]^ Mostly, the photoinduced segregation has been studied using photoluminescence spectroscopy, which detects the low‐bandgap phase very well as the electrons are likely to migrate into iodide‐rich regions and recombine there.^[^ ^16^ ^]^ However, it lacks quantitative information on the content of each phase, a limitation that can be addressed using XRD,^[^ ^30^ ^]^ though XRD does not reveal the bandgaps of the phases. TA spectroscopy bridges this gap by combining the capabilities of both techniques and has been successfully applied to study ion segregation.^[^ ^30^, ^31^, ^32^, ^33^, ^34^ ^]^

The triple‐cation mixed halide composition, (FA_0.76_MA_0.19_Cs_0.05_Pb(I_0.81_Br_0.19_)_3_ is commonly used in PSCs.^[^ ^35^ ^]^ However, ultrafast phenomena in this material have been rarely studied, especially compared to the standard MAPbI_3_ perovskite.^[^ ^4^, ^5^, ^6^, ^7^ ^]^ This work investigates intriguing photophysical processes in triple‐cation perovskites using ultrafast TA at various pulse fluences: charge cooling, charge recombination dynamics, and the observation of coherent acoustic phonons. Similarly, reports on ion segregation in such samples are scarce.^[^ ^26^, ^36^, ^37^ ^]^ To the best of our knowledge, there is no report on ion segregation study using ultrafast TA. A detailed experimental TA study was conducted to explore the basics of ion segregation in triple‐cation perovskites under ultrashort laser pulse irradiation. Systematic research was carried out to assess the impact of pulsed laser fluence on the kinetics and degree of ion segregation. Additionally, the effects of pulsed and continuous illumination, charge transport layers and contact materials were investigated in the perspective of ion segregation. The studied ultrafast phenomena and ion segregation are found to be strongly dependent on local heating effects originating from the thermalization of hot photoexcited carriers. This work may provide deeper insight into ultrafast electronic and acoustic processes taking place in perovskite devices, and shed more light on the fundamental mechanism of ion segregation observed in PSCs.

## 2 Experimental Section

A series of (FA_0.76_MA_0.19_Cs_0.05_Pb(I_0.81_Br_0.19_)_3_ PSCs were fabricated using the preparation and deposition procedure described before.^[^ ^38^ ^]^ In complete cells, compact TiO_2_ (deposited by spray pyrolysis at 450 °C using titanium diisopropoxide in ethanol) and mesoporous TiO_2_ (spin‐coated 30NR‐D, GreatCell Solar paste, diluted 1:6 w/w in EtOH) were used as electron transport layer (ETL) on FTO glass, while spiro‐OMeTAD (Sigma‐Aldrich) was used as hole transport layer (HTL) on the top of perovskite layer. Additionally, reference samples of only perovskite on glass, a sample without ETL (also on glass) and a sample without HTL were prepared. Samples were kept in the dark and in a dry atmosphere to avoid their degradation induced by moisture and light. A photograph of freshly prepared devices is shown in Figure S1a (Supporting Information). Figure S2a (Supporting Information) presents a cross‐section view of scanning electron microscopy for complete stack of PSC. The layers’ thicknesses are estimated to be as follows: FTO ≈500 nm, compact TiO_2_ –≈50 nm, mesoporous TiO_2_ – (150±20) nm, perovskite – (350 ±30) nm, spiro‐OMeTAD – (125±15) nm, gold – ≈100 nm. Effective perovskite thickness, including that mixed with mesoporous TiO_2_, is thus ≈400 nm. In one reference sample aluminum‐doped zinc oxide (AZO) of thickness ≈300 nm is deposited instead of gold.

The complete devices (each sample of size is 2.5 × 2.5 cm contained four devices which were measured independently) were characterized in terms of their PV performance through a mask of surface equal to 0.08 cm^2^. Current‐voltage characteristics were recorded using a potentiostat (model M101, Autolab) coupled to a photoelectric spectrometer, equipped with a solar simulator (Instytut Fotonowy, Poland). The simulator provides standard 1 Sun illumination using Xenon lamp and AM1.5G filter (calibrated by silicon solar cell 15 151, ABET). The stationary absorption spectra were obtained with a Jasco V‐770 spectrophotometer (with an integrating sphere).

TA spectroscopy studies in the transmission mode were performed using the setup delivered by Ultrafast Systems (Spectra‐Physics laser system and Helios spectrometer) with instrument response function (IRF) of 0.3–0.4 ps. Measurements were carried out in the 600–850 nm spectral range (corresponding to 1.46–2.07 eV on the energy scale) and temporal window of 3 ns, with 475 nm excitation. Such a wavelength, significantly lower than the material's bandgap, allows for selective investigation of the perovskite interfaces thanks to high absorbance in this range and thus, short penetration depth (≈50 nm). In some control experiments, 605 nm and 680 nm pump wavelengths were also used for which the penetration depth is much longer (≈150 and 200 nm, respectively). The pump pulse fluence was varied over broad range by changing pump pulse energy. E.g., pump pulse energy of 60 nJ corresponds to the fluence of 30 µJ cm⁻^2^ (the pump pulse spot has ≈0.5 mm diameterFull Width at Half Maximum ‐ FWHM) which, taking into account the 500 Hz pump repetition rate, gives the average irradiance equal to 15 mW cm⁻^2^ and the number of absorbed photons in the perovskite sample similar as that of ≈0.35 Sun continuous irradiation. LED system (Instytut Fotonowy) with monochromatic excitation chosen at 452 nm was used as a source of comparative continuous excitation. For the temperature effect studies, a sample was connected with a Peltier module working in heating or cooling mode. TA evolution in the fs‐ns domain was analyzed using Surface Explorer (Ultrafast Systems) software. A multi‐exponential function, convoluted with the IRF, was globally fitted to the kinetic vectors of selected singular values. This fitting provided the characteristic time constants and their corresponding wavelength‐dependent amplitudes, commonly referred to as pre‐exponential factor spectra or decay‐associated spectra. The temporal evolution of TA spectra over minutes and hours was globally fitted using OriginPro software (OriginLab Corporation) or ASUFIT software.^[^^]^ Figure S1b (Supporting Information) illustrates the beam configuration used in the TA experiment and depicts the concept of phase segregation occurring under illumination.

## 3 Results and Discussion

### 3.1 Solar Cell Performance

Upon the multiple device measurement, the average parameters of the devices were found to be as follows: open‐circuit voltage: 1.05 ± 0.02 V, short‐circuit current: 18 ± 1 mA cm⁻^2^, fill factor: 0.55 ± 0.02, which yields power conversion efficiency (PCE) above 10%. A relatively small fill factor is probably due to the large area of sputtered gold electrodes, while limited photocurrent is due to a slightly thinner perovskite layer (effective perovskite thickness of ≈400 nm, see exemplary SEM cross‐section picture in Figure S2a, Supporting Information and stationary absorption spectrum in Figure S2b, Supporting Information) than in the most efficient reports.^[^ ^40^ ^]^ Although the overall efficiency of device is relatively low compared to benchmark devices, the main reason behind it is the requirement of thin perovskite layer (≈400 nm) to perform TA experiments in the transmission mode. Moreover, based on the measured *J_sc_* of our samples and the stationary absorption spectra, we calculated the relative photocurrent, which is close to 100% (total APCE >90%).^[^ ^41^ ^]^ It indicates that charge separation efficiency is close to the maximum, so we confirm the good quality of our perovskite layer.^[^ ^41^ ^]^ Thus, the measurements of the photovoltaic parameters ensured us that the charge extraction rate constants are much higher than unwanted first order recombination rate constant. The primary aim of the work is to study ultrafast dynamics and ion segregation in the complete solar cell rather than the efficiency improvement in the device. Furthermore, electrochemical impedance spectroscopy measurements of the devices with varying bias and illumination intensity were performed under open circuit conditions.^[^ ^41^ ^]^ A detailed analysis is shown in Figure S3 (Supporting Information), depicting the near‐unity ideality factor which proves the good contact quality between perovskite and ETL/HTL layers.

### 3.2 Effect of Pump Fluence on the Ultrafast Dynamics

**Figure 1:**
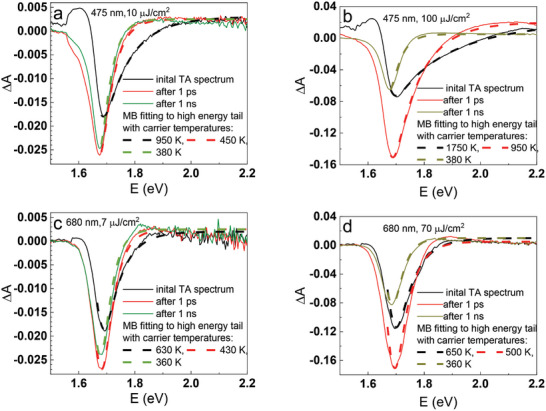
TA spectra for 475 nm pulse of two different pump fluences: a) 10 µJ cm⁻^2^ and b) 100 µJ cm⁻^2^ and for 680 nm pulse of two different pump fluences: c) 7 µJ cm^⁻2^ and d) 70 µJ cm^⁻2^. The high‐energy tail is fitted using the MB method to extract the hot carrier temperatures.

First, we present the TA dynamics in the time scale from IRF duration (≈300 fs) up to 3 ns for the complete solar cells probed in between the gold electrodes and pumped under different fluence from the glass (ETL) side. It should be noted that we used a very broad range of pump fluence varying by more than three orders of magnitude: from the smallest possible fluence of single µJ cm⁻^2^ (comparable to the fluence of the probing pulse) to the highest possible of several mJ cm⁻^2^ (when the permanent decomposition of perovskite was observed). The main excitation wavelength was set at 475 nm. Having in mind that the perovskite absorption onset is at 740 nm (Figure S2b, Supporting Information), such excitation provides an initial energy excess of ≈0.9 eV above the perovskite bandgap. **Figure** **1** presents exemplary spectra at selected pump‐probe time delays for two different pump pulse fluences. The high‐energy tail of TA bleach band spectra has been fitted using the Maxwell−Boltzmann (MB) method to extract the hot carrier temperatures (*T_c_*) from the equation:(1)^[^ ^6^, ^42^ ^]^

(1) $$\Delta A\left(E\right)\propto A_{0}\left(E\right)\exp\left(\frac{E_{F}-E}{k_{B}T_{C}}\right)$$

where *A_0_* (*E*) is stationary absorption as a function of energy.

As can be seen in Figure 1a,b, the pump energy excess corresponds to the initial temperature of the carriers *T_c_* ≈1000‐2000 K for different fluences. This energy is mainly used for local heating of the perovskite lattice (limited to the small pump spot surface). As a result, the local heating can be modified by varying pump fluence, since the number of photoexcited hot carriers is directly proportional to the fluence. The pump fluence significantly influences the charge decay dynamics, as nonlinear recombination processes are highly dependent on the population of photoexcited carriers (will be confirmed further). An essential aspect of our study is that the selected pump wavelength (475 nm) confines the initial excitation near the interface with the ETL, as the light penetration depth is ≈50 nm at this wavelength. The TA signals analyzed here are limited to the first 5 min of measurement at a fresh spot. Beyond this time, prolonged irradiation induces photoinduced ion segregation, altering the perovskite composition (which will be explored further in section 3.4).

The control experiments were also performed with 680 nm laser pulse excitation at several fluences. The excess energy in this case is only 0.15 eV (6 times less than with 475 nm pulses), so smaller *T_c_* and less local heating are expected than with 475 nm excitation. Indeed, as confirmed in Figure 1c,d, the fitted initial carrier temperatures are much lower, *T_c _* <700 K, and they do not vary as significantly with pump fluence as for the excitation with 475 nm pulses.

To obtain characteristic time constants, multi‐exponential global analysis was used to separate the average hot carrier cooling time constant (*τ_cool_*) from the charge recombination/extraction dynamics. The results with 475 nm excitation are shown in Figure S4 (Supporting Information) while **Table** **1** collects the obtained time constants (averaged from 2–5 measurements at the same fluence). The spectral feature of the first process shows the characteristic derivative shape of band‐edge shift (pre‐exponential factor spectra indicated in blue color in Figure S4, Supporting Information) due to charge screening or bandgap renormalization effect.^[^ ^43^, ^44^ ^]^ Following this cooling process, the TA spectrum of the perovskite evolves into a pronounced bleach, which is attributed to the band filling mechanism,^[^ ^9^, ^45^, ^46^ ^]^ This evolution is captured in the global fit as a second, slower‐time component with a negative amplitude and a minimum near the bandgap region (730–740 nm, red spectra in Figure S4, Supporting Information). For high pump fluences (above 30 µJ cm⁻^2^, corresponding to ≈10^19^ cm^−3^ initial carrier density) additional components are required in the global analysis to achieve satisfactory fit quality (green spectra in Figure S4, Supporting Information). These components display progressively shorter lifetimes and exhibit a blue shift in their spectra. This shift is attributed to the band filling effect, where higher‐energy states become occupied as the charge carrier population increases.^[^ ^6^, ^47^ ^]^ The examples of the fit quality are presented in Figure S5a,b (Supporting Information). It is important to note that the maximum possible excitation fluence we reached in the TA experiment was 4 mJ cm⁻^2^. At this fluence, rapid and permanent decomposition of the triple‐cation perovskite occurred within a single minute of excitation. This led to a reduction in the TA signal in the whole spectral range and damaged the perovskite layer (a bleached spot at the pump beam position). Figure S5c (Supporting Information) presents the effect of the decrease of the TA bleach amplitude over time and confirms that for the excitation at fluences smaller than 4 mJ cm⁻^2^ the decomposition of perovskite does not occur and the TA signal is stable over hours.

**Table 1:** TA dynamics and signal amplitude for different pump fluences at 475 nm.

Pump fluence, µJ/cm^2^	Charge cooling time *τ_cool_*, fs	Population decay time constants, ps	Bleach amplitude (sum of 2 components), ΔA
2	230	> 10000	0.005
9	300	6000	0.028
30	340	4600	0.050
30 (with Au)	420	160; 3300	0.050
45	460	180; 4200	0.085
65	510	100; 2800	0.110
100	620	50; 2000	0.120
200	680	44; 1500	0.200
400	960	33; 1300	0.250
600	1100	23; 1250	0.400
1000	1200	13; 1200	0.410
2000	1300	15; 1600	0.420
6000	900	27; 5400	0.100

As can be seen from Table 1, the charge cooling times gradually increase with higher pump fluence, from *τ_cool_* = 230 fs at 2 µJ cm⁻^2^ to *τ_cool_* = 1300 fs at 2 mJ cm⁻^2^. It can be noted that the smallest pump fluence used is comparable to the fluence in the probing light absorbed by the perovskite, so its further reduction is not meaningful. The typical explanation for a relatively slow hot carrier cooling process in perovskite and its dependence on the local heating and charge population is the hot phonon bottleneck effect.^[^ ^6^, ^7^, ^42^ ^]^ It is worth noting that, in principle slow cooling times could be utilized to extract hot carriers, potentially improving solar cell efficiency by overcoming the Shockley‐Queisser limit.^[^ ^6^, ^12^ ^]^ As for charge population decay, its dynamics depend on charge recombination and charge extraction to ETL/HTL coupled to charge diffusion.^[^ ^13^, ^14^, ^48^ ^]^ The bleach decay becomes faster with increasing pump fluence because of the increasing contribution of nonlinear recombination processes (due to the growing photoexcited charge population, *n*). E.g., as we have recently shown, at 30 µJ cm⁻^2^ the population decay is mainly governed by second‐order (radiative) recombination, but third‐order (Auger) recombination has also to be taken into account^[^ ^48^ ^]^ and its contribution increases at higher fluences (second and third order recombination rates are coupled to *n* ^2^ and *n* ^3^, respectively). It should be stressed that the time constants of population decay in Table 1 only show the time scales of the charge population decay process and do not have any physical meaning. To get the exact parameters of the charge extraction and recombination rate constants, a modeling including the charge diffusion in perovskite is necessary,^[^ ^14^, ^48^ ^]^ which was beyond the scope of the current work. Interestingly, both cooling time increase and population decay time decrease stop at very high pump fluence (above 1 mJ cm⁻^2^, Table 1). No further change in time constants is also accompanied by no further increment in the TA signal amplitude. We also conducted a test experiment by externally heating the sample to ≈50 °C using the Peltier device. Within experimental error, we have observed no significant effect on the bleach amplitude, charge cooling, and population decay time constants. However, we did notice a blue shift of ≈23 meV in the bleach peak when the temperature was increased to ≈50 °C, compared to room temperature conditions.

More importantly, TA experiments at 30 µJ cm⁻^2^ fluence were also performed through the gold electrodes of the PSC. Although the presence of gold reduced the probe pulse intensity, which consequently lowered the signal‐to‐noise ratio, the most important TA signal with the highest amplitude, ranging from 690 to 810 nm, was still successfully collected (Figure S4c, Supporting Information). Surprisingly, the time constants obtained from the global fit of the measurements with gold were similar to those acquired without gold at higher fluence (Table 1). E.g., the charge cooling time increased from 340 fs to 420 fs in the presence of gold, and the population decay times were in between those measured at 45 µJ cm⁻^2^ and 65 µJ cm⁻^2^ (roughly two times higher fluence). Notably, the amplitude of the TA signal was identical with and without gold (Figure S4b,c, Supporting Information), indicating that the charge population remained unchanged in both cases. We confirmed that the results through the gold layer were consistent under open circuit (electrodes not connected) and short circuit (electrodes connected) conditions. Based on our findings here, we propose that this unexpected behavior is likely due to the plasmonic effects of the thin (≈100 nm) gold layer, which enhance both local heating and the rates of nonlinear recombination.

Finally, the global analysis was also applied for the control TA experiments with 680 nm excitation and the results are shown in Table S1 (Supporting Information). For the corresponding TA signal amplitudes, *τ_cool_* is slightly faster than for 475 nm.^[^ ^47^ ^]^ Similar saturation appears as the bleach amplitude increases and the decay acceleration stops at high fluences as for 475 nm excitation.

The fluence‐dependent behavior described above has been previously demonstrated for perovskites,^[^ ^6^, ^47^ ^]^ but not for the benchmark triple cation mixed halide structure, especially across such a broad fluence range spanning three orders of magnitude. Furthermore, to the best of our knowledge, the unique behavior observed in complete devices with a gold layer is being reported here for the first time in any PSC.

### 3.3 Oscillations at High Local Heating

**Figure 2:**
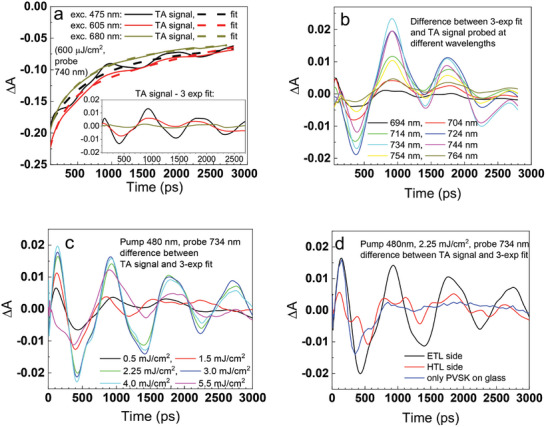
CLAP‐induced oscillations in TA signals. a) Effect of different pump wavelengths: original TA signal at bleach minimum and fitted 3‐exp function. The inset shows the extracted oscillation component as a difference between the TA signal and the fitted function. b) Effect of different probing wavelengths. c) Effect of different pump fluence. d) Effect of different sides of excitation and CLAP oscillations in bare perovskite layer.

At very high pump fluence ranging from 0.5 and 5 mJ cm⁻^2^, a remarkable feature appears in TA signals: long period (sub‐ns) oscillations in the bleaching kinetics with relatively long damping time (**Figure** **2**). Interestingly, within this fluence range, almost no significant changes in TA kinetics and amplitude are observed (Table 1). The observed oscillations can be attributed to the effect of coherent longitudinal acoustic phonons (CLAP).^[^ ^6^, ^49^, ^50^ ^]^ At certain conditions, a significant population of these phonons induces lattice strain, which alters refractive index. As this strain propagates through the material, it leads to the modulation of TA kinetics. Short‐period oscillations (from sub‐ps to few ps) in TA signals have been frequently reported for perovskite films, especially using femtosecond pulses with a duration significantly shorter than 100 fs (up to a few tens of fs).^[^ ^51^, ^52^, ^53^, ^54^ ^]^ These oscillations are typically attributed to coherent optical phonons generated directly from the cooling of hot carriers.^[^ ^6^, ^52^, ^55^ ^]^ On the contrary, acoustic phonons that are populated from optical phonons are rarely investigated using ultrafast spectroscopy. Under specific conditions, CLAP features were mainly observed in complicated transient reflection experiments for perovskite crystals and thin films.^[^ ^56^, ^57^, ^58^, ^59^, ^60^ ^]^ The reported oscillation periods varied typically from tens to hundreds of ps and the frequencies depended on both reflection angle and probing wavelength. As per our knowledge and primary literature survey, CLAP effects in transmission TA experiments on perovskites have almost not been reported so far, therefore we will describe the features of this effect in more detail since their properties are significantly different to those observed in transient reflection experiments.

Figure 2a presents the exemplary oscillations of the kinetics in bleach minimum. A multi‐exponential function was fitted to the kinetics and then subtracted from the experimental data to demonstrate the oscillations clearly for further analysis (e.g., inset in Figure 2a). The first feature of perovskite CLAP in transmission TA is presented in Figure 2a that compares the effect of excitations at 475, 605, and 680 nm. The results for both excitations show maxima and minima at the same time delays, but the oscillation amplitude is significantly higher for shorter excitation wavelengths. It can be probably explained by the fact that the pump pulse at 475 nm generated coherent phonons near the interface, while the pump at 605 nm and 680 nm wavelengths have a penetration depth approximately three and four times greater (≈150 and 200 nm, respectively). Hence, the propagating strain lattice is more stretched with a smaller peak intensity. Moreover, as shown in the previous section, a longer excitation wavelength brings a lower initial hot carrier temperature. Next, we compared the amplitude of the oscillation for different probing wavelengths (Figure 2b). The maximum oscillations are observed at the bleach minimum (734 nm) and they decrease on both sides of the bleach, with a reduction of ≈10 times at 30 nm away from the minimum on both sides. This behavior suggests the feature reported for other semiconductors where CLAP amplitude is proportional to the derivative of the stationary absorption with respect to the energy (wavelength).^[^ ^50^, ^61^ ^]^ Hence, the oscillations have the highest amplitude for those wavelength at which the absorption onset has the steepest slope, which corresponds to the minimum of the bleach in TA. Another interesting feature is that the maxima and minima are at the same time delays for all probing wavelengths at which the oscillations are detected. In many reports from transient reflection measurements, the CLAP oscillations caused a shift in the bandgaps, such that the maxima on the short‐wavelength side corresponded to the minima on the long‐wavelength side and vice versa. Our results indicate a different impact on the TA signal in transmission mode, since the amplitude of the bleach increases and decreases simultaneously on both sides (its “breathing” can be also visualized in the color 2D picture of TA – Figure S6a, Supporting Information). It can be also noted that the amplitude and period of the oscillations do not depend on the angle at which the sample is measured (the angle between the probe beam and the sample front surface – Figure S6b, Supporting Information).

Typically, the amplitude of the CLAP oscillations is reported to increase with the pump fluence.^[^ ^6^, ^62^ ^]^ Our results partially confirm this behavior, but above certain fluence threshold, there is no further increase in the amplitude. As can be seen in Figure 2c, the highest amplitude of the oscillations is between 2 and 3 mJ cm⁻^2^. At 4 or 5.5 mJ cm⁻^2^, the amplitude starts to decrease, which can be correlated with the lowering of the amplitude of the TA bleach signal itself. Further fluence increment is not possible. As mentioned earlier, the perovskite material undergoes permanent decomposition rapidly. It can also be noted that the damping of the oscillations (decrease of its amplitude over time) is slightly faster for higher fluence (e.g., compare the signals at 3 and 4 mJ cm⁻^2^ in Figure 2c). The period of oscillations for the investigated samples was *T*≈900 ps, which was deduced from both the distances between the maxima or minima as well as from FFT analysis of the TA signal (Figure S6c, Supporting Information), which reveals the peak at frequency 1/*T* = 1.1 MHz. The speed of the sound *v* at which the CLAP strain is propagating along the perovskite layer can be calculated according to the simple formula:^[^ ^6^, ^49^, ^57^ ^]^

(2) $$v=\frac{4d}{T}$$

where *d* is the thickness of the perovskite layer (400 nm), and *T* is the period of oscillations. Thus, in the case of triple cation perovskite, the speed of sound yields the value of ≈1800 m^−1^s, which is in good agreement with the reports for other perovskites.^[^ ^6^, ^55^, ^60^, ^62^ ^]^ We also tested the effect of the excitation from different sides and found that the excitation from the HTL side results in a significantly lower amplitude of the oscillations than that from the ETL (glass) side (at the same fluence, Figure 2d). The FFT analysis reveals that the oscillations are not purely sinusoidal and likely contain additional components, such as the second harmonic of the main period. This effect is more pronounced when excitation is applied from the HTL side, showing additional modulations to the signal (Figure 2d).

We also examined how the CLAP oscillations appear in the TA measurements of a pure perovskite layer on glass, as opposed to the full cell discussed so far. Surprisingly, in the case of pure perovskite, the damping is so strong that only the first maximum and minimum are observable, as shown in Figure 2d. Such fast damping has also been reported in the only previous work on CLAP observation in perovskites using transmission TA.^[^ ^62^ ^]^ Most likely, reflections of the acoustic phonons from air and glass are much weaker than those from ETL and HTL. In the case of full cells, the oscillation amplitude decreases by only about two times after three periods (at ≈2800–3000 ps delay), suggesting that the strain can propagate several times in the perovskite, bouncing back and forth at the contact layers.

Finally, our results indicate that CLAP fingerprints in TA of perovskite, measured in the most common transmission mode appear under very specific conditions: the pump fluences should be high (close to the perovskite damage threshold), short excitation wavelength, and full cells (with ETL and HTL layers) are required for slow damping time. Most probably, it is the reason why such oscillations have not been reported so far. Thus, the possibility of their detection using a relatively popular time‐resolved technique opens the potential for broader studies of acoustic phonons in perovskites. In particular, for precisely determined thicknesses of the perovskite layer, the methodology proposed in our work enables the way to determine the speed of sound in different perovskites.

### 3.4 Photoinduced Ion Segregation

**Figure 3:**
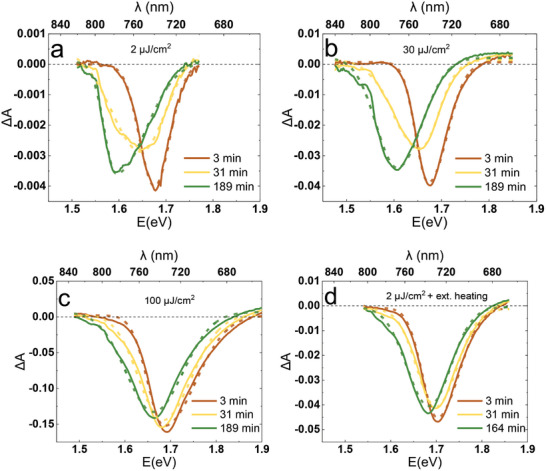
Changes observed during pulsed laser irradiation for the recorded TA spectra (solid lines) and independently fitted asymmetric Gaussian curves (equation (3), dashed lines) for pulse fluence: a) 2 µJ cm⁻^2^, b) 30 µJ cm⁻^2^, c) 100 µJ cm⁻^2^ and d) 30 µJ cm⁻^2^ with external heating applied.

**Figure** **3** presents the change in the bleach band (solid line) observed in the complete device irradiated from the ETL side between the gold electrodes (in open‐circuit mode) with a pulsed laser of varying pump fluence. The pump‐probe delay was chosen at 2 ps for the reasons presented below. The peak of this band shifts to a lower energy and stabilizes after ≈3 h, which is associated with the formation of a new, iodide‐rich phase. Importantly, the transition state between the initial and the final shape of the band is characterized by lower amplitude and greater width. This suggests that the change arises from the superposition of two bleaches (one decaying and another rising) rather than a continuous shift of one bleaching signal. Referring to the nature of anion segregation‐related TA changes observed previously in a different perovskite composition,^[^ ^33^ ^]^ it can be confirmed that these changes are a result of a superposition of two TA spectra arising from two distinct phases of different bandgap: the initial, decaying mixed phase and the emerging iodide‐rich phase of a lower bandgap. As the bandgaps of the two phases differ by a small value in our case (because of the already high concentration of iodide, 81%, in the initial composition), the two bleaching bands appear to be merged (and the apparent bleach band evolution is observed).

#### 3.4.1 TA Kinetics before and after Segregation

First, we briefly present the analysis of ultrafast TA kinetics for the above sample excited at 30 µJ cm⁻^2^ (Figure 3b). We will compare the results at the start of irradiation (when no segregation is present) and after 3 h of pulsed irradiation (by which point, as demonstrated below, the majority of the sample has undergone segregation). The global analysis was approximated by a double‐exponential fit (see Section 3.2), and the exemplary results are shown in Figure S7 (Supporting Information). As discussed in section 3.2, the first component describes the initial charge cooling process. Its time constant increases from 0.33 ± 0.02 ps at the beginning to 0.42 ± 0.04 ps after 3 h of irradiation. The second component represents the average decay of the bleach band and corresponds to the average lifetime of the photoexcited charge population. The time constant associated with the second component decreases from 5.5 ± 0.5 ns to 3.0 ± 0.5 ns after 3 h of irradiation. This indicates that, following segregation, the charge population lifetime of the iodide‐rich phase is nearly half that of the initial mixed phase (i.e., the second‐order recombination rate constant approximately doubles), while charge cooling in the segregated phase slows down by ≈20–30%. It is important to note that for a perovskite sample on glass (where almost no segregation occurs, as shown in Figure S9, Supporting Information), no significant changes in the time constants are observed. These findings validate our selection of a 2 ps time delay for analyzing TA spectra that evolve over minutes under pulsed irradiation. At shorter time delays, the charge cooling process remains incomplete, while at longer time delays, the amplitude of TA spectra is influenced not only by the ion segregation, but also by the variation in charge population lifetime. Finally, a short time after excitation also permits observation of the process close to the interfaces, as charge diffusion occurs on the time scale of tens and hundreds of picoseconds.^[^ ^14^, ^48^ ^]^

#### 3.4.2 Pulse Fluence and External Heating Influence

The effect of varying pump pulse fluence (from 2 to 100 µJ cm⁻^2^) will be described by how the TA spectra at 2 ps evolve during irradiation. The influence of beam intensity on the ion segregation can be easily observed, with all spectra evolutions shown in Figure S8 (Supporting Information) and selected ones presented in Figure 3a–c. First, it is noticeable that decreasing the pulse fluence from 30 µJ cm⁻^2^ to lower values has a minimal impact on the extent of ion segregation. In contrast, increasing the fluence significantly alters the nature of the transition. E.g., at 100 µJ cm⁻^2^, the final bleaching band (recorded at 189 min, Figure 3c) is only slightly shifted toward lower energies compared to the initial bleaching (at 3 min), in contrast to the shifts observed under weaker illumination conditions. The plots also show that bands are generally wider under stronger illumination. However, this is an effect caused by band‐filling in perovskites and broadening of the bleach band (shown in section 3.2) at short pump‐probe delays with an increased number of excited charges^[^ ^47^ ^]^ and is not related to ion segregation.

In the first, simple analysis, the TA bleach bands of the perovskite were fitted using the asymmetric Gaussian peak function, further denoted as AG. The function is characterized by different width parameters on each side of the center using the following equations:

(3) $$AG\;\left(E,\;E_{c},\;h,\;w_{L},\;w_{H},\;y\right)=y+h\cdot e^{-0.5{\left(\frac{E-E_{c}}{w_{L}}\right)}^{2}}\;\left(E<E_{C}\right)$$

(4) $$AG\;\left(E,\;E_{c},\;h,\;w_{L},\;w_{H},\;y\right)=y+h\cdot e^{-0.5{\left(\frac{E-E_{c}}{w_{H}}\right)}^{2}}\;\left(E\geq E_{C}\right)$$

where *E* stands for energy (independent variable), *E_c_* is the position of the peak center, *h* is the height of the curve, and *y* is the offset of the function. *w_L_* and *w_H_* represent the two widths of the curve fitted to the bleach band, corresponding to the low‐energy (L) and high‐energy (H) sides of the peak center, respectively. This approach ensures the required asymmetry of the Gaussian function, which is essential for achieving a quality fitting with fair accuracy. Figure 3a–c demonstrates the changes which are observed during pulsed laser irradiation for the recorded TA spectra (fitted AG curves are marked with dashed lines). In the analysis, the *E_C_* parameter allows us to monitor the process of ion segregation, with the value decreasing as the segregation proceeds. Importantly, the *E_C_* values obtained this way are free of the effects of possible beam power fluctuations, which could affect the signal amplitude.

**Figure 4:**
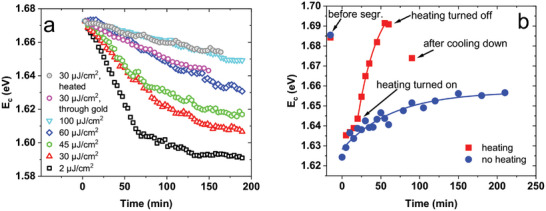
a) Peak position (E_c_) shifts obtained from independent asymmetric Gaussian fitting using equation (3) for selected measurements, showing the decreased ion segregation rate for increased pump fluence, for an externally heated sample, and a sample measured through a gold electrode; data sets were shifted vertically to start at one point. b) Peak position shift during perovskite regeneration in the dark, with heating versus without heating.

The bleaching spectra collected for all the laser intensities and illumination times were fitted with AG curves, and resulted peak position parameter *E_C_* was then plotted as a function of time. **Figure** **4**a depicts the peak shift plots for selected measurements, enabling a comparison of the rate of anion segregation. It can be observed that increased pulsed laser irradiation power influences the ion segregation process, with segregation being notably reduced under higher pulse fluence. These results might appear to be counter‐intuitive. If segregation happens under the influence of light, why would an increased amount inhibit it? However, it has been reported that higher temperature induces the re‐mixing of anions in the lattice^[^ ^63^ ^]^ or slows down the segregation process.^[^ ^29^ ^]^ We notice that due to the short wavelength of the pump beam compared to the material's absorption edge (475 nm vs 740 nm), strong local heating occurs. A significant portion of incoming energy is thermalized, and transferred to the crystal phonons,^[^ ^6^ ^]^ as demonstrated in the previous sections. Therefore, when the beam power is high (the number of hot carriers is large), the local temperature rises, and the increased thermal energy of anions provokes the mixing of ions, competing with the segregating influence of photoexcited charge carriers. Subsequently, the segregation phenomenon becomes slower and of reduced extent. The local heating effect may account for the contradictory reports regarding the influence of irradiation intensity on the ion segregation process.

To confirm the temperature influence on the reduced ion segregation under increased irradiance, a solar cell was measured under 30 µJ cm⁻^2^ pulse fluence, and externally heated to ≈50 °C near the beam spot location using the Peltier device. Interestingly, the change in TA spectrum during 30 µJ cm⁻^2^ irradiation with heating resembles the one obtained for the highest beam powers (without external heating) described in the previous section. Figure 3d presents the initial, transitional, and final spectra for the heated sample, and a similarity of the peak evolution with the high fluence spectra (Figure 3c) can be noticed. As Figure 4a shows, the peak shift for the heated sample at 30 µJ cm⁻^2^ follows approximately the same path as in the case of high pulse fluence (100 µJ cm⁻^2^). These observations confirm that effects observed for increased pump pulse energies can be associated with the local heating of the sample. Moreover, it suggests that an increase in the local temperature between 30 µJ cm⁻^2^ and 100 µJ cm⁻^2^ pulse irradiation is equal to ≈30 K (the differences between ≈50 °C and room temperature).

In another experiment, a sample was illuminated with 680 nm laser pulses of changed fluence. As mentioned in section 3.2, the excess energy in this case is 6 times smaller than with 475 nm pulses, so we expect significantly lower local heating and thus, weaker influence on ion segregation dynamics. As shown in Figure S9a (Supporting Information), the differences between the peak shifts were much smaller, which provides additional support for the local heating origin of ion segregation inhibition. The reason for a smaller segregation at 680 nm is that the penetration depth at 680 nm is ≈4 times longer, resulting in weaker influence from a TiO_2_ interface for 680 nm than for 475 nm excitation.

Interestingly, almost no segregation was observed in perovskite/glass sample, while for samples lacking HTL or ETL, its extent was reduced as compared to a complete PV device (as shown in Figure S9b,c, Supporting Information). This observation highlights the crucial role of charge transport layers in ion segregation and further supports our choice of complete cell for the investigation. As we ensure the conditions for most profound segregation, any changes in its dynamics can be measured with greater accuracy and lower signal‐to‐noise ratio.

#### 3.4.3 Perovskite Regeneration Study

As mentioned above, it has been reported that elevated temperature leads to an increased rate of perovskite regeneration as it promotes the re‐mixing of ions.^[^ ^63^ ^]^ Therefore, another experiment was performed to verify how increased temperature affects the regeneration of the triple cation perovskite in the dark. First, the regeneration was monitored for a sample that was irradiated for 3 h, while it was kept at room temperature during the entire regeneration time. Accordingly, the same sample was then irradiated for 3 h in another place and after a couple of minutes, heating was turned on. It was turned off an hour after the start of the measurement and then the sample was cooled down. Figure 4b illustrates the shift of the bleach band peak obtained from AG fitting during the regeneration with and without heating. It is visible that when heating is turned on (red points), the curve strongly diverges from the non‐heated regeneration curve (blue points). The curves were fitted with mono‐exponential functions and we obtained a time constant of 65 ± 13 min and amplitude of 29 ± 2 meV for the non‐heated curve and a time constant of 27 ± 4 min and amplitude of 65 ± 4 meV for the heated curve. Although a small part of this amplitude might be due to the temperature effects on the bleach position (described in section 3.2), it still confirms that increased temperature makes regeneration of the perovskite both faster and to a greater extent. Figure 4b shows that the difference between the final (1.656 eV) and initial (1.685 eV) peak position is 29 meV for the non‐heated sample while in the case of heated perovskite, the difference (final, after cooling down: 1.674 eV, initial: 1.685 eV) is just 11 meV, ≈3 times lower. Thus, comparing the final states at the same temperature (after cooling down the sample heated during regeneration), in the experiment without heating the perovskite reached only half of its way toward complete regeneration, while with heating it is ≈80%.

#### 3.4.4 Continuous versus Pulsed Illumination and Measurement through Gold

**Figure 5:**
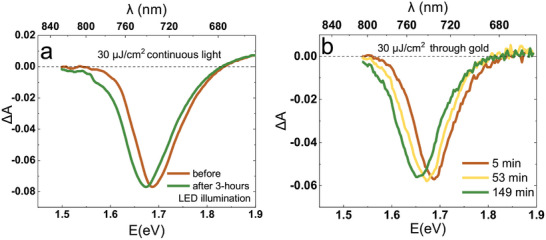
a) Bleaching band shift observed during an illumination with continuous LED light. b) Sample excited with 30 µJ cm⁻^2^ and measured through gold electrodes.

To assess the significance of light coming in short pulses instead of continuous irradiation, a solar cell was illuminated with a monochromatic LED light of 452 nm wavelength, with average irradiance on the device equating to our reference conditions (15 mW cm⁻^2^). With the cooling‐mode Peltier device, the sample was kept at a temperature not higher than 24 °C. After 3 h of illumination, only a slight redshift of the TA spectrum was observed (**Figure** **5**a), with a peak shift of 13 meV, compared to 66 meV in the experiment with 30 µJ cm⁻^2^ pulsed illumination (Figure 3b). This experiment shows that short pulses of high peak power induce much more ion segregation than continuous light of comparable average irradiance. It was reported that the segregation process is driven by charge carriers accumulated on grain boundaries or at the interfaces and by the electric fields connected with those charges.^[^ ^24^ ^]^

Grain boundary effects are not important in the case of our studied sample, because we do not observe any significant ion segregation without ETL/HTL layers (Figure S9b,c, Supporting Information). According to our calculations (the details shown in Supporting Information), the electric field in mesoporous TiO_2_ can be ≈3 orders of magnitude higher during a few microseconds after each pump pulse comparing to continuous irradiation using LED or 1Sun simulator. Such time scale should be enough to initiate bromide/iodide exchange. We have also calculated (Figures S10–12 and additional discussion in Supporting Information) that the average number of photogenerated charges (e.g., over 1 s) is similar under both TA pulsed conditions and under 1Sun illumination.^[^ ^64^ ^]^ However, at short periods after each pulse excitation the number of photogenerated carriers can exceed the one under photo‐stationary conditions by several orders of magnitude, leading to very high electric field after charge injection into the contact layers on a short time scale.

Therefore, one can put forward a hypothesis that high charge carrier concentration over a short time (induced by the laser pulse) creates forces that can efficiently displace the anions, inducing phase separation. These forces might be related to the high local electric field created by the electrons injected during a short time into mesoporous TiO_2_ that interpenetrates the perovskite material close to the ETL side. The preliminary results indicate that when the mesoporous TiO_2_ layer is replaced with a compact SnO_2_ layer yielding a similar photocurrent, then ion segregation is suppressed (Figure S13, Table S2, and additional discussion in Supporting Information). It could be noticed that the charge mobility in TiO_2_ is lower than in SnO_2_, so the relatively high population of electrons injected into TiO_2_ (after pulse excitation of perovskite) can stay there for a sufficiently long time for the halides to move under the created electric field. The backward movement of ions during the period when the sample is not illuminated is relatively slow. Therefore, a complete reversal of this process will not happen before the following laser pulse excites the charges and induces the segregation again. To observe the ion segregation under continuous illumination in triple‐cation perovskite, much higher continuous irradiance might be required. This observation is consistent with previous reports of an energy threshold required to initiate ion segregation.^[^ ^37^ ^]^

Finally, a complete device was also measured through gold electrodes using the pump fluence of 30 µJ cm^2^. The results are shown in Figures 4a and 5b. From Figure 4a, and by comparing Figures 3b and 5b, it can be observed that the peak shift associated with the ion segregation is significantly reduced in the presence of gold, compared to the measurement in the area outside an electrode. Therefore, the presence of gold in the vicinity of the excited region makes ion segregation slower or to a lesser extent. Regarding the probable influence of the gold layer on ion segregation dynamics, a couple of hypotheses can be proposed. The gold layer may affect the local temperature in the sample, eventually leading to a reduction of ion segregation. It could be speculated that gold, being a good heat conductor, would lead to efficient heat dissipation, which should not impede ion segregation. However, one might also consider plasmonic effects occurring in the gold layer, which could elevate the local temperature, as discussed in section 3.2. Another possible explanation could be that the gold electrode facilitates faster neutralization of charges accumulated on the spiro‐OMeTAD layer, thereby weakening the electric forces that drive ion segregation. To verify which mechanism operates and isolate the plasmonic and electronic effects due to metal electrode, we conducted an experiment on a solar cell using a non‐metal like, aluminum‐doped zinc oxide (AZO) as the electrode, instead of gold. It was found that halide segregation occurs in the cells with AZO electrodes to much higher extent than those with gold electrodes (Figure S9d, Supporting Information). This suggests that plasmonic effects, which enhance local heating, are likely responsible for the suppression of ion segregation in the area of gold electrodes.

#### 3.4.5 Global Fitting with an Extended Model

The way of data analysis presented above, based on tracking the bleaching band peak shift, allows for a qualitative comparison between the ion segregation rates in various measurements. However, it provides little quantitative data regarding the nature of processes taking place in the material. To achieve more insight into the details of the anion segregation process the values of the following three parameters that can govern the ion segregation process should be established:

- *τ_T_* – transition time constant – describing the average time constant of the evolution from the mixed phase into the segregated phases;
- *D_S_* – degree of segregation – as previously reported in the literature,^[^ ^65^ ^]^ the mixed perovskite does not undergo segregation fully. In our case, this parameter describes the fraction of the material that ultimately divides into separate phases (bromide‐rich and iodide‐rich, of which only the latter is visible on TA spectra);
- *ΔE_C_* – bandgap difference between mixed and iodide‐rich phase. This parameter can be associated with iodide concentration in the iodide‐rich phase (the higher the iodide concentration, the bigger the bandgap difference).

To calculate the three parameters mentioned above in cases where two bleaching spectra overlap, making the analysis more challenging, we propose a new fitting model, with a detailed description provided in the Supporting Information. In brief, the procedure begins by determining *τ*_T_ through the global mono‐exponential fitting of TA spectra. Then, *D_S_* and *ΔE_C_* are determined based on an assumption that two phases contribute to the recorded TA spectra: the original mixed‐anion phase and the developing iodide‐rich phase. Therefore, the resultant spectrum is described by the double asymmetric Gaussian function (called double‐AG).

**Figure 6:**
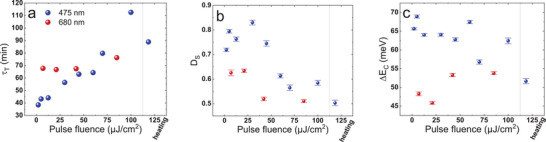
Parameters of the phase segregation process as functions of pulse fluence for 475 and 680 nm excitation wavelength, with the values for the externally heated sample included; a) transition time constant obtained from global fitting of exponential functions to signal dynamics; b) degree of segregation and c) bandgap difference, both obtained from global fitting of the double‐AG function.

In this study, we confirmed that the signal dynamics can be approximated very well by a mono‐exponential function fitted to the data, with the fitting correlation coefficient *r* ^2^ exceeding 0.99 in most cases, and always greater than 0.98. **Figure** **6**a presents the values of the globally fitted *τ_T_* parameter. As observed, for 475 nm pump wavelength, increasing the pulse fluence results in a nearly linear increase in the time constant, ranging from ≈40 min at 2 µJ cm⁻^2^ to over 110 min at 100 µJ cm⁻^2^. This indicates that the process slows down as the light intensity increases. For 680 nm pump, this effect is not visible in a significant way (Figure 6a). Figures 6b and 6c show the global double‐AG fitting results for the degree of segregation (*Ds*) and the bandgap difference, respectively. For 475 nm, *D_S_* is close to 0.8 for lower fluences (80% of the perovskite undergoes segregation); however, the value drops significantly beyond the pulse fluence of 45 µJ cm⁻^2^, reaching below 0.6 (under 60% of the perovskite undergoes segregation) for the highest fluences (Figure 6b). For 680 nm, we also observe a minor reduction in *D_S_*. When it comes to the bandgap difference, a slight decline in *ΔE_C_* was observed for 475 nm at higher pulse fluences (Figure 6c) though a slight increment was found for 680 nm. However, the amount of data is not sufficient to establish clearly whether it is a significant relationship. The results (for 475 nm excitation) obtained for the heated sample give similar results to those for the highest investigated pulse fluences (70–100 µJ cm⁻^2^). This fact, together with the finding that segregation time constant (*τ_T_*) varies less with fluence for lower excess energy (680 nm vs 475 nm, Figure 6a) further supports the hypothesis that the observed suppression of ion segregation for the increased pulse fluence is caused by the heat coming from the thermalization of excited charge carriers.

## 4 Conclusions

A comprehensive analysis of various phenomena occurring during the excitation of triple‐cation mixed halide perovskites with ultrashort laser pulses is presented. TA experiments using 100 fs pump pulses at 475 nm (≈0.9 eV above the perovskite bandgap) were conducted with pump fluences varying over more than three orders of magnitude (from 2 µJ cm⁻^2^ to 6 mJ cm⁻^2^). These experiments, which resulted in initial hot carrier temperatures reaching several thousand kelvins, revealed a range of effects associated with varying local heating and charge population densities. Upon increasing the pump fluence, the carrier cooling time constant increases from ≈200 fs to ≈1300 fs, while the charge population decay time constant decreases from tens of ns to tens of ps. The presence of gold electrode in the complete cells increases the local heating effect and nonlinear recombination rate. At fluences in the single mJ/cm^2^ range, the aforementioned time constants cease to vary, and the oscillation in the TA signal emerges due to CLAP. Further increase in pump fluence leads to permanent changes (decomposition) in perovskite material.

Several key features of CLAP oscillations in TA signals measured in transmission mode are observed: the oscillation amplitude peaks at the highest TA bleach signal and increases with shorter excitation wavelengths, remaining unaffected by the probe angle relative to the sample surface. The CLAP phase is independent of pump/probe wavelengths, pump fluence, and probing angle. Damping time constants are relatively slow (several ns) in complete PSCs with charge‐extracting layers, unlike in pure perovskite layers on glass. These features allow easy determination of CLAP frequency in complete cells using a TA setup, which can be used, e.g., to determine the sound velocity in perovskite (≈1800 m^−1^s in this case).

Furthermore, this study offers new insights into ion segregation in a widely used triple cation perovskite, which had not been extensively analyzed before with TA. The detailed analysis confirmed the occurrence of ion segregation under pulsed laser illumination, despite the high initial iodide concentration (0.81). The timescale (40–110 min) is consistent with literature reports.^[^ ^37^ ^]^ TA spectroscopy proved to be a powerful tool for studying segregation effects in perovskites, tracking phase transitions via bleaching band evolution. We observed that the higher pump intensity inhibits anion segregation, likely due to laser‐induced local heating, which has been confirmed by similar effects in externally heated samples. Moreover, another TA experiment revealed that ion segregation is minimal or absent in the triple‐cation perovskite under continuous light illumination at the same wavelength and average irradiance, potentially explaining the scarcity of reports on this effect in the material. These observations suggest that the peak irradiance and the resulting high density of excited charge carriers, rather than average irradiance, are crucial for ion segregation. The presence of a gold electrode also inhibits ion segregation, possibly due to increased local heating from plasmonic effects. The control experiments at 680 nm excitation (≈0.15 eV above the perovskite bandgap, 4 times less than at 475 nm) confirmed the key role of local heating in the ion segregation suppression and CLAP generation.

We also propose a new method to gain more insight into ion segregation when two TA bleaching spectra overlap. A double asymmetric Gaussian fitting model provides high‐quality quantitative data, enabling tracking of parameters like transition time constant, segregation degree, and bandgap difference between phases.

## Conflict of Interest

The authors declare no conflict of interest.

## Supporting information



Supporting Information

## Data Availability

The data that support the findings of this study are available from the corresponding author upon reasonable request.

## References

[1] A. Kojima , K. Teshima , Y. Shirai , T. Miyasaka , J. Am. Chem. Soc. 2009, 131, 6050. 19366264 10.1021/ja809598r

[2] “Best Research‐Cell Efficiency Chart,” can be found under https://www.nrel.gov/pv/cell‐efficiency.html.

[3] S. Sahare , H. D. Pham , D. Angmo , P. Ghoderao , J. MacLeod , S. B. Khan , S.‐L. Lee , S. P. Singh , P. Sonar , Adv. Energy Mater. 2021, 11, 2101085.

[4] S. A. Kulkarni , T. Baikie , P. P. Boix , N. Yantara , N. Mathews , S. Mhaisalkar , J. Mater. Chem. A 2014, 2, 9221.

[5] C. Qiu , L. Wagner , J. Liu , W. Zhang , J. Du , Q. Wang , Y. Hu , H. Han , EcoMat 2023, 5, e12268.

[6] J. Fu , S. Ramesh , J. W. Melvin Lim , T. C. Sum , Chem. Rev. 2023, 123, 8154. 37276018 10.1021/acs.chemrev.2c00843 PMC10347435

[7] V. Ravali , T. Ghosh , Chem. Commun. 2023, 59, 13939. 10.1039/d3cc04297a 37934456

[8] X. Chen , P. V. Kamat , C. Janáky , G. F. Samu , ACS Energy Lett. 2024, 9, 3187. 38911533 10.1021/acsenergylett.4c00736 PMC11190987

[9] T. C. Sum , N. Mathews , G. Xing , S. S. Lim , W. K. Chong , D. Giovanni , H. A. Dewi , Acc. Chem. Res. 2016, 49, 294. 26820796 10.1021/acs.accounts.5b00433

[10] Z. Yuan , M. Yang , L. Zhang , W.‐G. Li , T. Tian , H. Pang , J. Phys. Chem. C 2024, 128, 20720.

[11] H. Pang , S. Du , J. Deng , W. Kong , Y. Zhao , B. Zheng , L. Ma , Small 2024, 20, 2401797. 10.1002/smll.202401797 38577831

[12] Z. Guo , Y. Wan , M. Yang , J. Snaider , K. Zhu , L. Huang , Science 2017, 356, 59. 28386007 10.1126/science.aam7744

[13] L. M. Herz , Annu. Rev. Phys. Chem. 2016, 67, 65. 26980309 10.1146/annurev-physchem-040215-112222

[14] J. Leng , J. Liu , J. Zhang , S. Jin , J. Phys. Chem. Lett. 2016, 7, 5056. 27973883 10.1021/acs.jpclett.6b02309

[15] C. C. Boyd , R. Cheacharoen , T. Leijtens , M. D. McGehee , Chem. Rev. 2019, 119, 3418. 30444609 10.1021/acs.chemrev.8b00336

[16] E. T. Hoke , D. J. Slotcavage , E. R. Dohner , A. R. Bowring , H. I. Karunadasa , M. D. McGehee , Chem. Sci. 2015, 6, 613. 28706629 10.1039/c4sc03141e PMC5491962

[17] G. F. Samu , C. Janáky , P. V. Kamat , ACS Energy Lett. 2017, 2, 1860. 10.1021/acsenergylett.4c00736 PMC11190987 38911533

[18] C. A. Aranda , A. O. Alvarez , V. S. Chivrony , C. Das , M. Rai , M. Saliba , Joule 2024, 8, 241.

[19] H. Choe , D. Jeon , S. J. Lee , J. Cho , ACS Omega 2021, 6, 24304. 34604614 10.1021/acsomega.1c03714 PMC8482395

[20] M. C. Brennan , A. Ruth , P. V. Kamat , M. Kuno , Trends Chem. 2020, 2, 282.

[21] S. Draguta , O. Sharia , S. J. Yoon , M. C. Brennan , Y. V. Morozov , J. S. Manser , P. V. Kamat , W. F. Schneider , M. Kuno , Nat. Commun. 2017, 8, 200. 28779144 10.1038/s41467-017-00284-2 PMC5544754

[22] M. C. Brennan , S. Draguta , P. V. Kamat , M. Kuno , ACS Energy Lett. 2018, 3, 204.

[23] A. J. Knight , A. D. Wright , J. B. Patel , D. P. McMeekin , H. J. Snaith , M. B. Johnston , L. M. Herz , ACS Energy Lett. 2019, 4, 75.

[24] R. A. Belisle , K. A. Bush , L. Bertoluzzi , A. Gold‐Parker , M. F. Toney , M. D. McGehee , ACS Energy Lett. 2018, 3, 2694.

[25] A. J. Knight , L. M. Herz , Energy Environ. Sci. 2020, 13, 2024.

[26] F. Ruf , P. Rietz , M. F. Aygüler , I. Kelz , P. Docampo , H. Kalt , M. Hetterich , ACS Energy Lett. 2018, 3, 2995.

[27] J. R. Vicente , J. Chen , J. Phys. Chem. Lett. 2020, 11, 1802. 31995980 10.1021/acs.jpclett.9b03734 PMC8409127

[28] P.‐K. Kung , M.‐H. Li , C.‐F. Lin , P. Chen , J. Mater. Chem. C 2024, 12, 11181.

[29] A. D. Wright , J. B. Patel , M. B. Johnston , L. M. Herz , Adv. Mater. 2023, 35, 2210834. 10.1002/adma.202210834 36821796

[30] A. J. Barker , A. Sadhanala , F. Deschler , M. Gandini , S. P. Senanayak , P. M. Pearce , E. Mosconi , A. J. Pearson , Y. Wu , A. R. Srimath Kandada , T. Leijtens , F. De Angelis , S. E. Dutton , A. Petrozza , R. H. Friend , ACS Energy Lett. 2017, 2, 1416.

[31] S. J. Yoon , S. Draguta , J. S. Manser , O. Sharia , W. F. Schneider , M. Kuno , P. V. Kamat , ACS Energy Lett. 2016, 1, 290.

[32] S. J. Yoon , M. Kuno , P. V. Kamat , ACS Energy Lett. 2017, 2, 1507.

[33] J. T. DuBose , P. V. Kamat , J. Am. Chem. Soc. 2020, 142, 5362. 32083862 10.1021/jacs.0c00434

[34] J. Tang , W. Tian , F. Sun , Q. Sun , J. Leng , S. Zhao , S. Jin , J. Phys. Chem. Lett. 2023, 14, 2800. 36907991 10.1021/acs.jpclett.3c00332

[35] C. Dong , Z.‐K. Wang , L.‐S. Liao , Energy Technol. 2020, 8, 1900804.

[36] D. Di Girolamo , N. Phung , F. U. Kosasih , F. Di Giacomo , F. Matteocci , J. A. Smith , M. A. Flatken , H. Köbler , S. H. Turren Cruz , A. Mattoni , L. Cinà , B. Rech , A. Latini , G. Divitini , C. Ducati , A. Di Carlo , D. Dini , A. Abate , Adv. Energy Mater. 2020, 10, 2000310.

[37] Z. Andaji‐Garmaroudi , M. Abdi‐Jalebi , D. Guo , S. Macpherson , A. Sadhanala , E. M. Tennyson , E. Ruggeri , M. Anaya , K. Galkowski , R. Shivanna , K. Lohmann , K. Frohna , S. Mackowski , T. J. Savenije , R. H. Friend , S. D. Stranks , Adv. Mater. 2019, 31, 1902374. 10.1002/adma.201902374 31489713

[38] K. Pydzińska‐Białek , G. Nowaczyk , M. Ziółek , Chem. Mater. 2022, 34, 6355.

[39] E. Katilius , J. Hindorff , N. Woodbury , “ASUFIT program,” can be found under, https://www.public.asu.edu/~laserweb/asufit/asufit.html.

[40] M. Saliba , J.‐P. Correa‐Baena , C. M. Wolff , M. Stolterfoht , N. Phung , S. Albrecht , D. Neher , A. Abate , Chem. Mater. 2018, 30, 4193.

[41] K. Pydzińska‐Białek , V. Drushliak , E. Coy , K. Załȩski , J. Flach , J. Idígoras , L. Contreras‐Bernal , A. Hagfeldt , J. A. Anta , M. Ziółek , ACS Appl. Mater. Interfaces 2020, 12, 30399. 32515941 10.1021/acsami.0c07095 PMC7497635

[42] Y. Yang , D. P. Ostrowski , R. M. France , K. Zhu , J. van de Lagemaat , J. M. Luther , M. C. Beard , Nat. Photonics 2016, 10, 53.

[43] M. T. Trinh , X. Wu , D. Niesner , X.‐Y. Zhu , J. Mater. Chem. A 2015, 3, 9285.

[44] F. Deschler , M. Price , S. Pathak , L. E. Klintberg , D.‐D. Jarausch , R. Higler , S. Hüttner , T. Leijtens , S. D. Stranks , H. J. Snaith , M. Atatüre , R. T. Phillips , R. H. Friend , J. Phys. Chem. Lett. 2014, 5, 1421. 26269988 10.1021/jz5005285

[45] S. Narra , E. Jokar , O. Pearce , C.‐Y. Lin , A. Fathi , E. W.‐G. Diau , J. Phys. Chem. Lett. 2020, 11, 5699. 32609524 10.1021/acs.jpclett.0c01589

[46] J. S. Manser , P. V. Kamat , Nat. Photonics 2014, 8, 737.

[47] K. Pydzińska‐Białek , J. Szeremeta , K. Wojciechowski , M. Ziółek , J. Phys. Chem. C 2019, 123, 110.

[48] K. Szulc , K. Pydzińska‐Białek , M. Ziółek , Materials 2023, 16, 7110. 38005040 10.3390/ma16227110 PMC10672245

[49] N. K. Tailor , P. Maity , S. Satapathi , J. Phys. Chem. Lett. 2022, 13, 5260. 10.1021/acs.jpclett.2c01369 35674417

[50] C. Thomsen , J. Strait , Z. Vardeny , H. J. Maris , J. Tauc , J. J. Hauser , Phys. Rev. Lett. 1984, 53, 989.

[51] T. Debnath , D. Sarker , H. Huang , Z.‐K. Han , A. Dey , L. Polavarapu , S. V. Levchenko , J. Feldmann , Nat. Commun. 2021, 12, 2629. 33976185 10.1038/s41467-021-22934-2 PMC8113605

[52] P. Guo , Y. Xia , J. Gong , D. H. Cao , X. Li , X. Li , Q. Zhang , C. C. Stoumpos , M. S. Kirschner , H. Wen , V. B. Prakapenka , J. B. Ketterson , A. B. F. Martinson , T. Xu , M. G. Kanatzidis , M. K. Y. Chan , R. D. Schaller , Adv. Funct. Mater. 2020, 30, 1907982.

[53] J. Fu , M. Li , A. Solanki , Q. Xu , Y. Lekina , S. Ramesh , Z. X. Shen , T. C. Sum , Adv. Mater. 2021, 33, 2006233. 10.1002/adma.202006233 33576093

[54] M. Scholz , M. Morgenroth , K. Oum , T. Lenzer , J. Phys. Chem. C 2018, 122, 5854.

[55] J. Fu , Q. Xu , G. Han , B. Wu , C. H. A. Huan , M. L. Leek , T. C. Sum , Nat. Commun. 2017, 8, 1300. 29101381 10.1038/s41467-017-01360-3 PMC5670184

[56] P. Guo , C. C. Stoumpos , L. Mao , S. Sadasivam , J. B. Ketterson , P. Darancet , M. G. Kanatzidis , R. D. Schaller , Nat. Commun. 2018, 9, 2019. 29789666 10.1038/s41467-018-04429-9 PMC5964251

[57] C. Thomsen , H. T. Grahn , H. J. Maris , J. Tauc , Phys. Rev. B 1986, 34, 4129. 10.1103/physrevb.34.4129 9940178

[58] Z. Y. Zhang , G. P. Wang , Adv. Funct. Mater. 2023, 33, 2214542.

[59] P.‐A. Mante , C. C. Stoumpos , M. G. Kanatzidis , A. Yartsev , Nat. Commun. 2017, 8, 14398. 28176755 10.1038/ncomms14398 PMC5309855

[60] P. Maity , J. Yin , B. Cheng , J.‐H. He , O. M. Bakr , O. F. Mohammed , J. Phys. Chem. Lett. 2019, 10, 5259. 31434482 10.1021/acs.jpclett.9b02100

[61] G. S. Kanner , Z. V. Vardeny , B. C. Hess , Phys. Rev. B 1990, 42, 5403. 10.1103/physrevb.42.5403 9996122

[62] F. Sun , B. Wu , S. Jin , Chin. Opt. Lett. 2022, 20, 100010.

[63] T. Elmelund , B. Seger , M. Kuno , P. V. Kamat , ACS Energy Lett. 2020, 5, 56.

[64] H. P. Parkhomenko , M. M. Solovan , S. Sahare , A. I. Mostovyi , D. Aidarkhanov , N. Schopp , T. Kovaliuk , M. Kaikanov , A. Ng , V. V. Brus , Adv. Funct. Mater. 2024, 34, 2310404.

[65] K. Suchan , J. Just , P. Beblo , C. Rehermann , A. Merdasa , R. Mainz , I. G. Scheblykin , E. Unger , Adv. Funct. Mater. 2023, 33, 2206047.

