# Supplementary material for: Insights Into the Local Heating Effects in Triple‐Cation Mixed‐Halide Perovskite Cells: Charge Dynamics, Coherent Phonons, Anion Segregation
Source: Small. 2025 Jan 16;21(8):2408541. doi: 10.1002/smll.202408541 (PMC11855263; doi:10.1002/smll.202408541)
Supplement: Supplementary file 1 — Supporting Information [file SMLL-21-2408541-s001.pdf]

NANO · MICRO
small

Supporting Information

for *Small*, DOI 10.1002/smll.202408541

Insights Into the Local Heating Effects in Triple-Cation Mixed-Halide Perovskite Cells: Charge Dynamics, Coherent Phonons, Anion Segregation

*Jacek J. Baranowski, Sanjay Sahare, Mykhailo Solovan, Patryk Florczak and Marcin Ziółek**

Supporting Information

for

Insights into the Local Heating Effects in Triple-Cation Mixed-Halide Perovskite Cells: Charge Dynamics, Coherent Phonons, Anion Segregation

Jacek J. Baranowski[1], Sanjay Sahare[1], Mykhailo Solovan[1], Patryk Florczak[2], Marcin Ziółek[1 *]

[1]*Faculty of Physics and Astronomy, Adam Mickiewicz University, Poznan 61-614, Poland*

[2]*NanoBioMedical Centre, Adam Mickiewicz University, Poznan 61-614, Poland*

**Corresponding Authors:* marcin.ziolek@amu.edu.pl

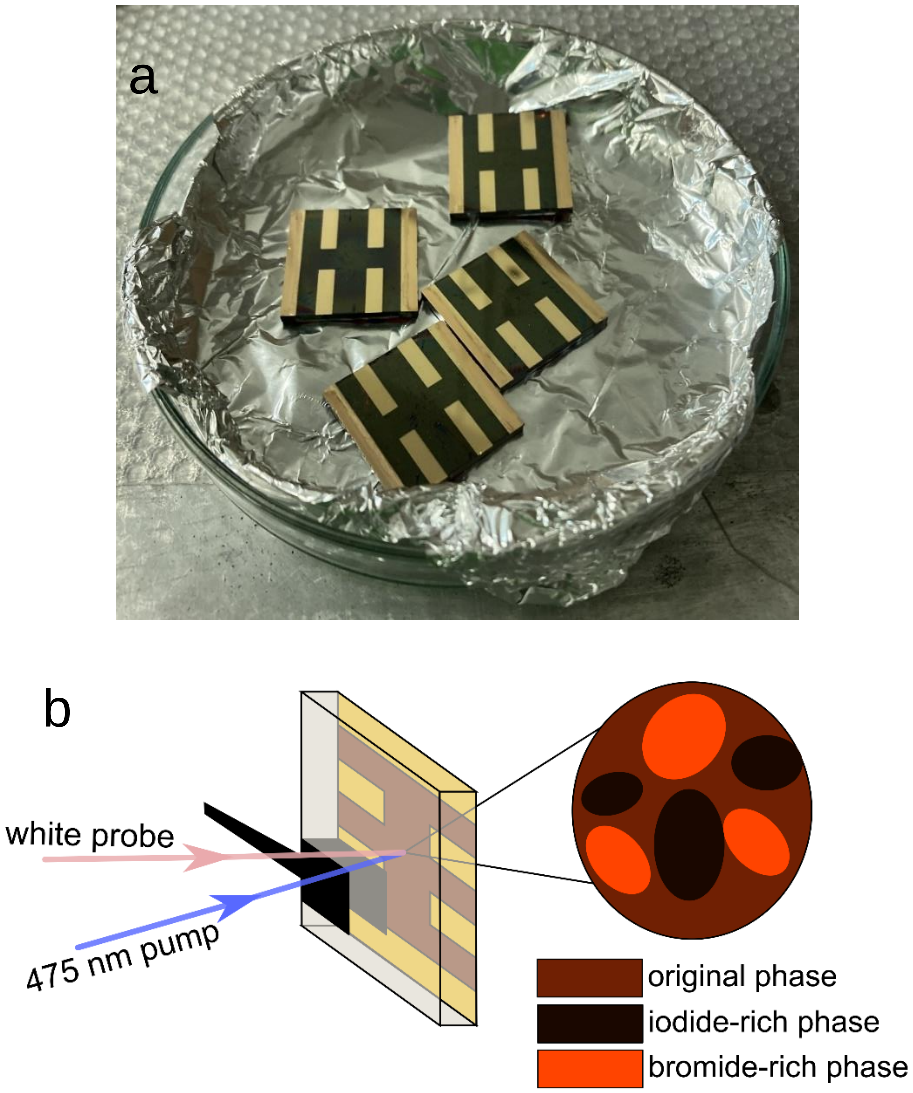

Figure S1. *(a) The triple-cation solar cells shortly after preparation, with gold electrodes deposited. (b) A scheme of the beam configuration in the TA experiment with the investigated sample placed in a holder. Both pump and probe beams penetrate the sample from the ETL (TiO_2) side. As a result, segregated phases appear in the sample, which can be seen on TA spectra.*

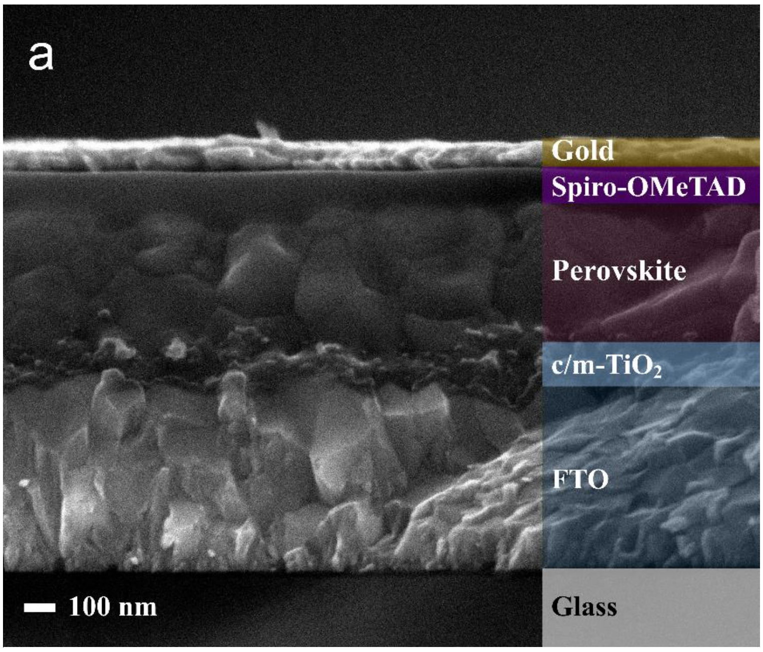

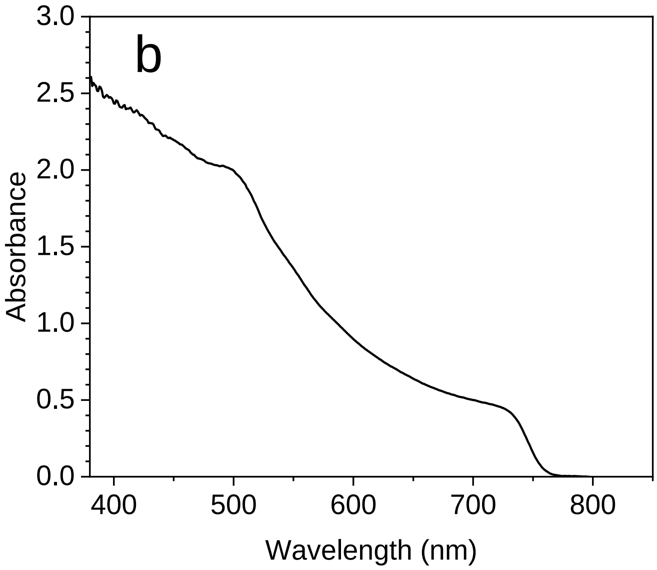

Figure S2. *(a) Cross-section of a solar cell studied through SEM, with layers labeled. "TiO_2" includes both the compact and the mesoporous layer. (b) Typical stationary absorption spectrum of a triple-cation perovskite solar cell. The absorption edge is visible around 740 nm (1.68 eV) indicating approximate bandgap value.*

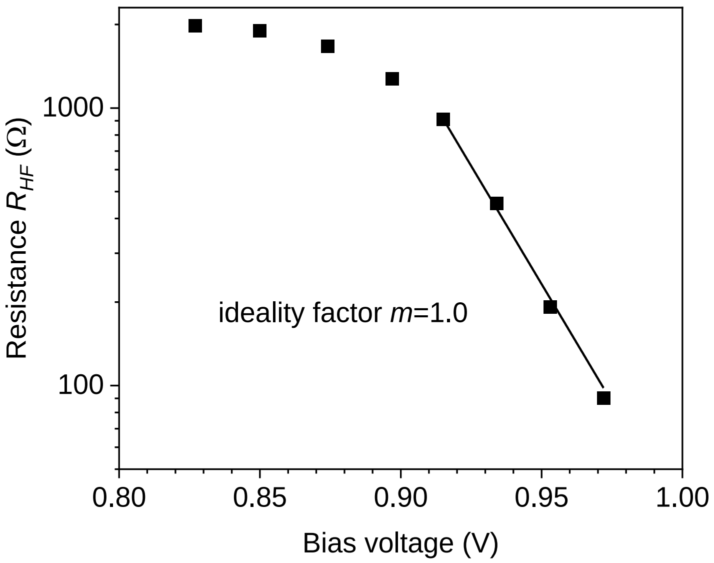

Figure S3. *Resistance of high-frequency arc analyzed from EIS data measured at different biases in open circuit conditions of the exemplary studied solar cell. The ideality factor m is calculated from the slope of the graph of the high-frequency resistance (R_{HF}) vs. bias potential (V) according to the following equation: $R_{HF}(V) = R_{0HF}\ exp(-eV/mk_BT)$, where R_{0HF} is the resistance at bias potential equal to zero, k_B is the Boltzmann constant, and T is temperature.*[1] *The solid line shows the fit around the V_{OC} of the cell with the fit quality of r^2=0.998. The calculated ideality factor is m=1.0. The resistance is shown in the logarithmic scale.*

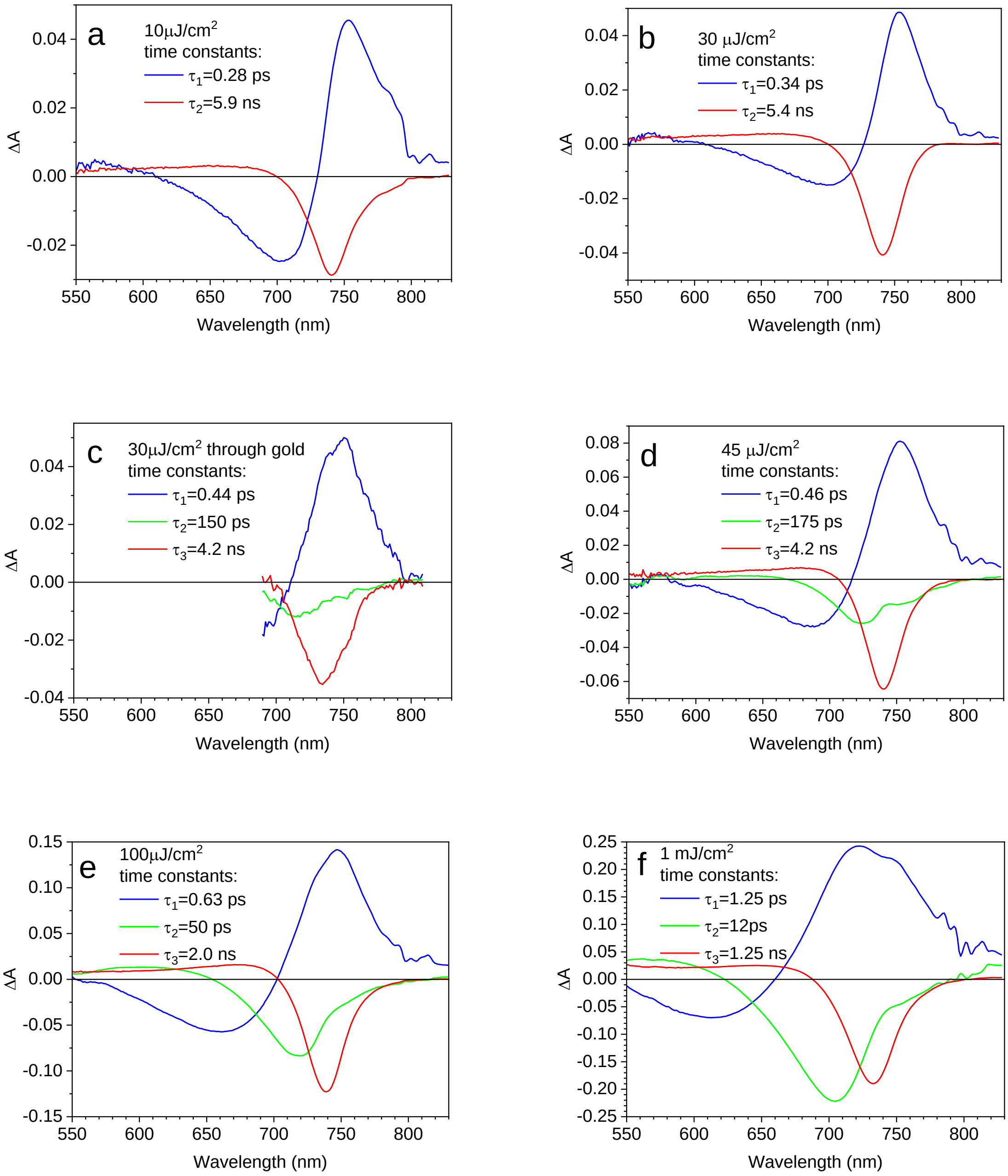

Figure S4. *Global TA fit results (wavelength-dependent amplitudes associated with the indicated fitted time constants of two- or three-exponential function convoluted with IRF) at selected different pump fluences.*

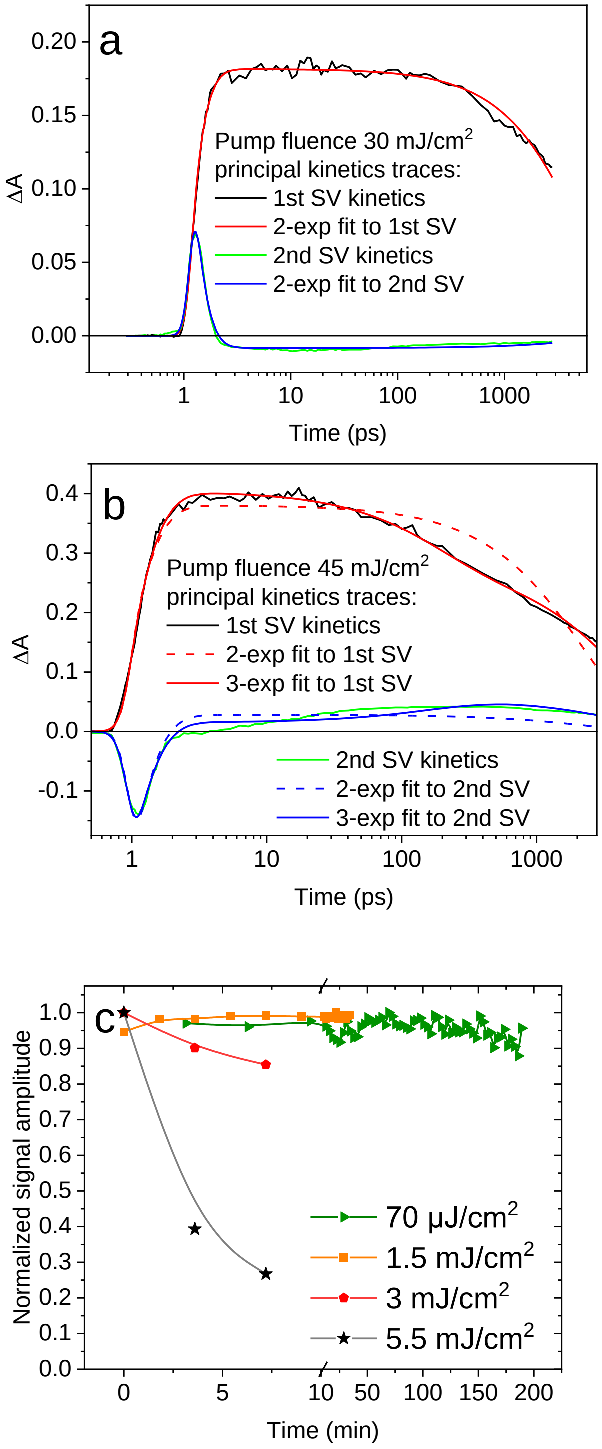

Figure S5. *(a-b): Examples of the global fit quality (2- or 3-exp fit to first two singular value – SV - kinetics). (c): TA signal stability at different pump fluences at 475 nm. The probing wavelength was chosen at which the ion segregation over time has no influence (close to the isosbestic points from Figure 3).*

Table S1. *TA dynamics and signal amplitude for different pump fluences at 680 nm.*

Pump fluence, μJ/cm^2	Charge cooling time τ_{cool}, fs	Population decay time constants, ps	Bleach amplitude (sum of 2 components), ΔA
7	300	5700	0.026
21	370	740; 3300	0.065
42	460	83; 2200	0.130
70	500	8; 1700	0.170
100	600	6.6; 1200	0.260
600	1000	6.9; 1200	0.370
4000	1000	10; 1600	0.340

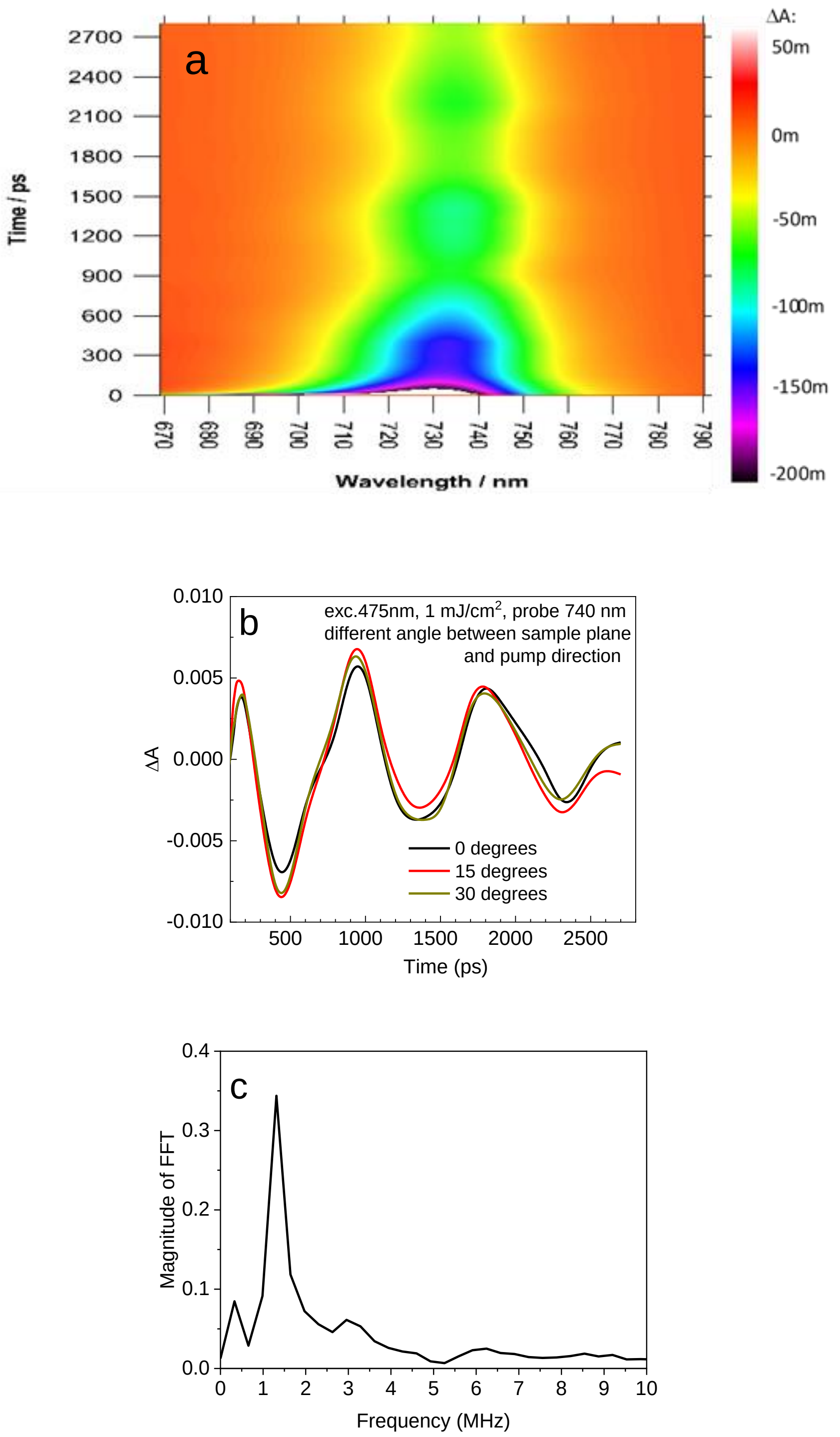

Figure S6. *(a) Color 2D picture of exemplary TA data collected for 2 mJ/cm^2 pump fluence (at 475 nm) showing CLAP oscillations.(b) No effect of varying the degree between pump pulse direction and sample plane; the plots show the difference between TA amplitude and 3-exp fit. (c) Example of FFT analysis of the subtracted oscillation part for 2 mJ/cm^2 pump fluence (at 475 nm).*

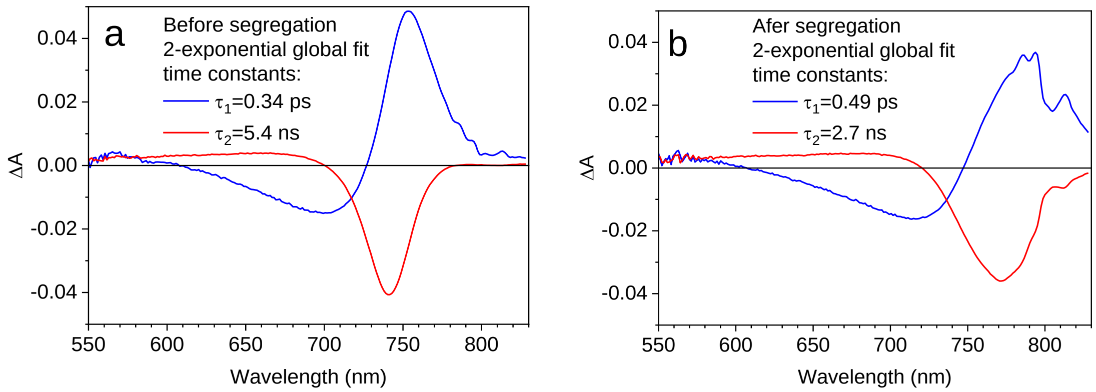

Figure S7. *Example of double-exponential global fit (wavelength-dependent amplitudes associated with the indicated time constants) of TA data at the beginning (a) and after (b) ion segregation in the complete device after excitation pump fluence 30 μJ/cm^2.*

Figure S8. *(continued on next page): Solid lines: changes in recorded TA spectra of complete perovskite devices recorded for varying pulse fluence and for the externally heated sample. Dashed lines: globally fitted curves obtained with the double-AG model.*

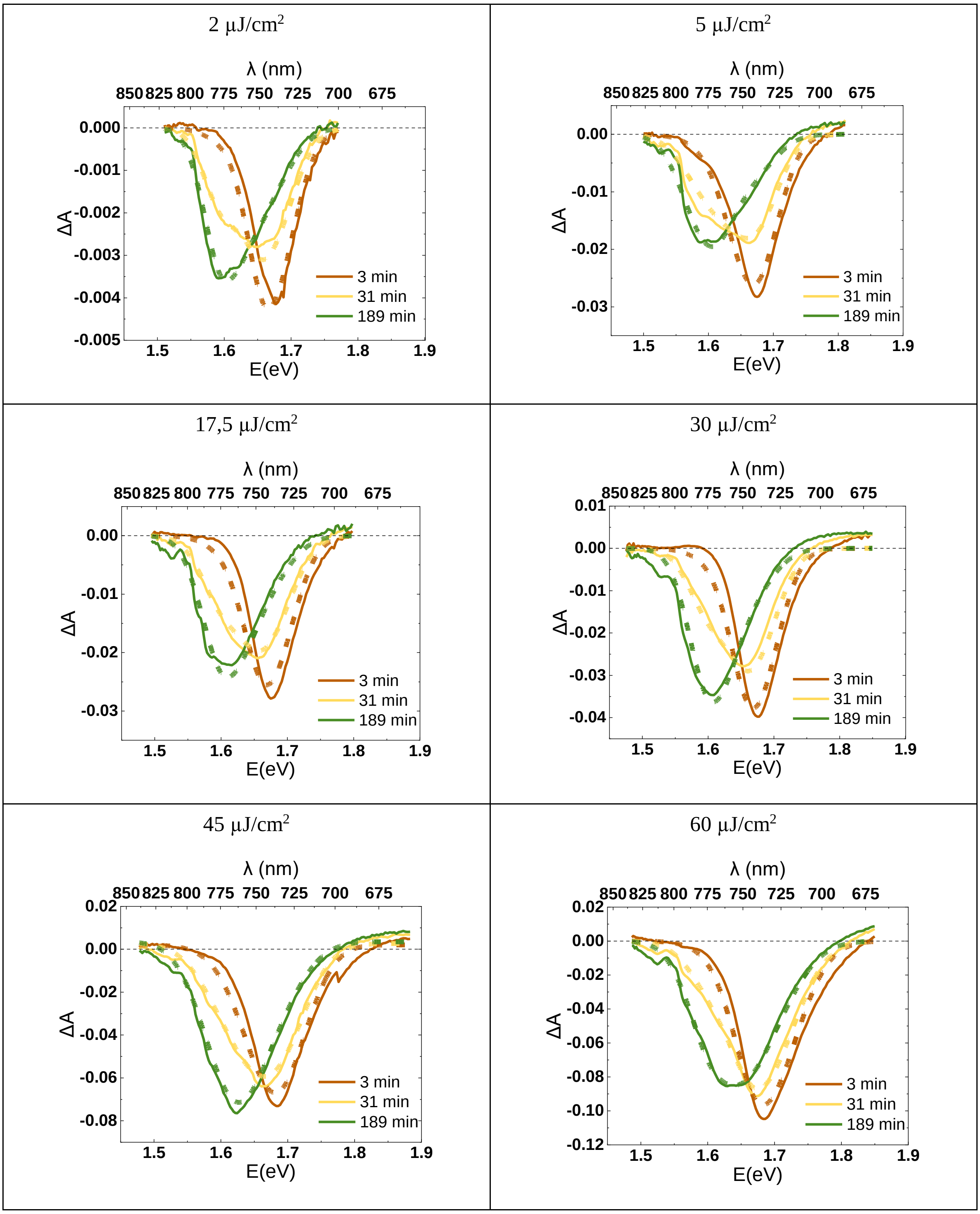

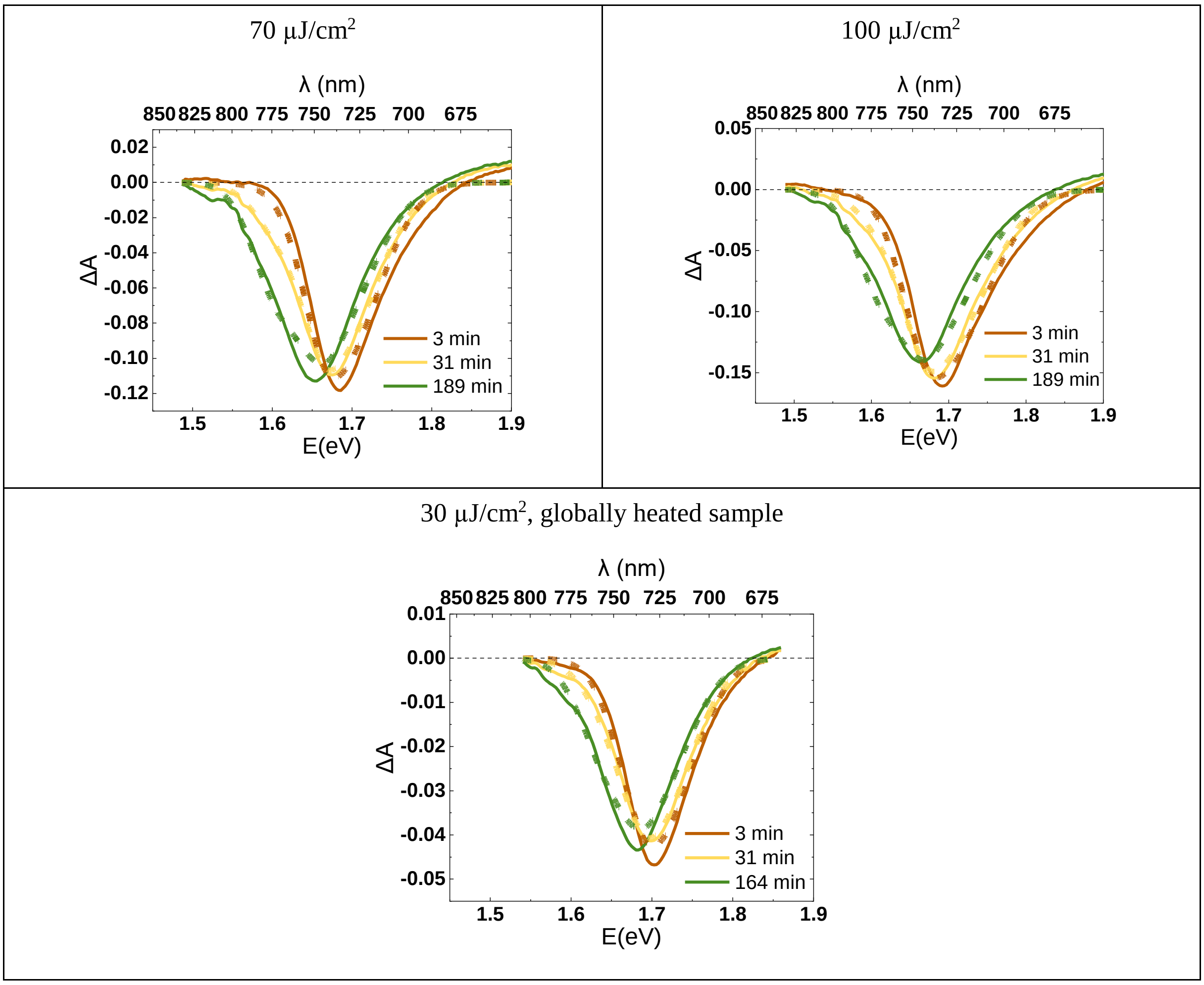

Figure S8 *- continuation*

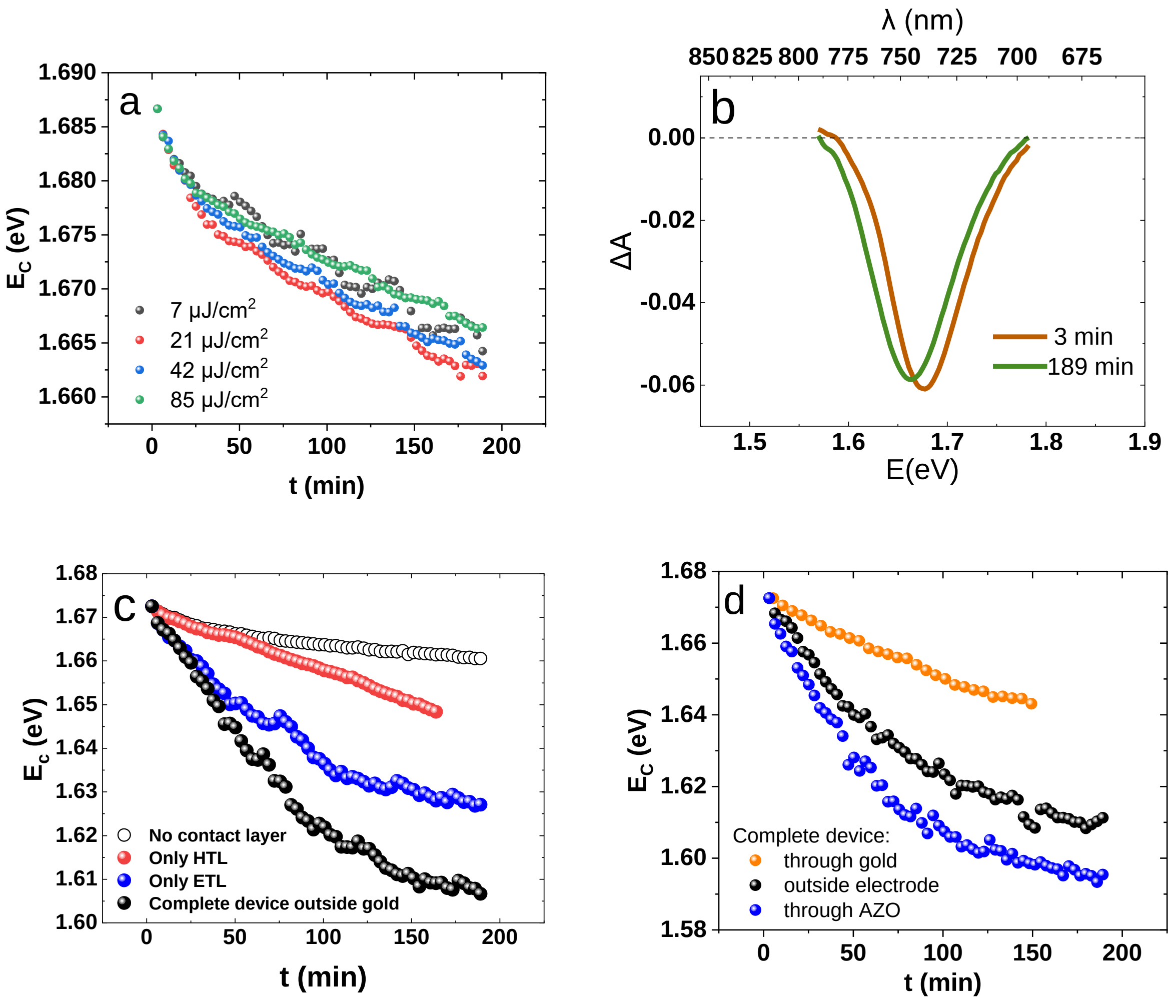

Figure S9. *(a) Effect of the excitation at 680 nm at different fluences. (b) Effect of irradiation of the reference sample on glass. (c) Effect of contact layers. (d) Effect of non-metallic AZO electrode compared with the gold electrode solar cell. The excitation was 475 nm with fluence 30 μJ/cm² for (b-d). It can be observed in (d) that the segregation with AZO is slightly higher than in the region without AZO which can be attributed by the improved heat dissipation offered by AZO layer.*

Estimation of the electric field difference under 1Sun and under pulsed laser excitation

For the pump pulse fluence of I=30 μJ cm^{-2} at λ=475 nm (the typical conditions for observation of halide segregation) the density of photons (per irradiated surface) is:

$$n_{ph} = \frac{I\lambda}{hc} = 7.2 \times 10^{13}\ \mathrm{cm}^{-2}.$$

The perovskite absorbance at 475 nm is A≈2, which means that almost all (99%) photons are absorbed and converted into electron-hole pairs. At the given fluence, all photoexcited charges either recombine or are extracted to ETL/HTL on the time scale of Δt≈10 ns.[2] Moreover, for I=30 μJ/cm^{2,} the dominant channels of charge depopulation are second and third-order recombination, whereas, only ~ 3% of the photoexcited electrons are injected into ETL.[2] It means that during Δt≈10 ns the electron flux that reaches ETL can be estimated as:

$$F_{pulse} = 3\%\ \text{of}\ \frac{n_{ph}}{\Delta t} = 1.4 \times 10^{20}\ \ \mathrm{s}^{-1}\mathrm{cm}^{-2}.$$

This electron flux present in ETL is responsible for the high local electric field that can drive ion segregation. The average time during which electrons diffuse through ETL can be calculated as:

$$\tau_{diff} = \frac{d^2}{D_0},$$

where d – is the thickness of the layer and D_0 is the diffusion coefficient. The diffusion coefficient is very low, D_0≈10^{-4} cm^2 s^{-1} for a TiO_2 mesoporous layer (d=150nm).[3] Therefore The time during which electrons remain in TiO_2, generating an electric field, is relatively long (τ_{diff} ≈ 3 μs), which is sufficient to allow at least a single halide exchange event in the perovskite material.

On the contrary, during constant 1Sun (AM1.5) illumination, the flux of absorbed photons calculated based on the absorption spectra of the perovskite layer is equal to N_{ph}=1.2×10^{17} s^{-1}cm^{-2}, obtained from integration of absorption spectra of perovskite multiplied by photon flux for AM 1.5 G.[4] Since the relative photocurrent is close to 100% (*total_APCE*>90%), most of the photoexcited electron-hole pairs are injected into contact materials and the contribution of first-order (trap-assisted) recombination can be neglected. Thus, the electron flux at 1Sun conditions is equal to $F_{1Sun} \approx N_{ph}$=1.2×10^{17} s^{-1}cm^{-2}, which is 3 orders of magnitude less than F_{pulse}. Therefore, at least on the time scale of single μs, the electric field from electrons in TiO_2 is ~1000 times stronger for pulse excitation than under 1Sun. This could likely explain why halide segregation in triple cation perovskite observed under continuous irradiation (Figure 5a) is much smaller compared to the segregation observed under pulsed excitation during TA experiments (Figure 3).

Modeling of charge generation rate under 1Sun and under pulsed laser excitation

The charge carriers' generation under 1Sun illumination rate has been calculated using the transfer matrix simulation of the optical field distribution within the entire device stack by considering the thicknesses, extinction coefficient *k(λ)*, and refraction index *n(λ)* of all functional layers.[5,6] Absorption spectra for the perovskite active layer were experimentally measured (see Figure S2b), and those for other commonly used functional layers were obtained from the literature.[5,7–11]

Position and wavelength-dependent generation rate *G(x, λ)* and integrated value of *G(x)* over all wavelengths within the perovskite active layer under AM1.5G illumination conditions are shown in Figure S10 and Figure S11.

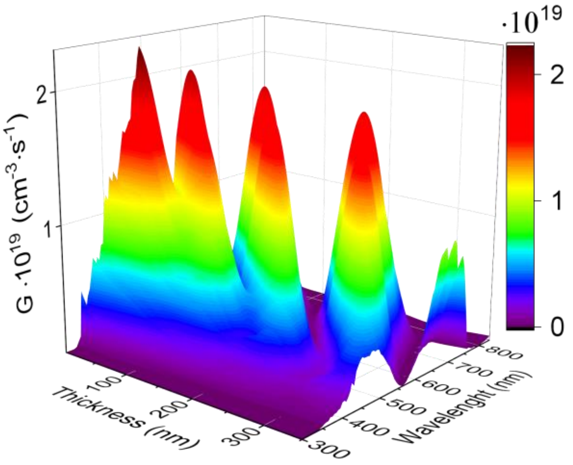

Figure S10. *Generation rates G(x, λ) depending on the wavelength and position in the active layer, for AM 1.5 illumination.*

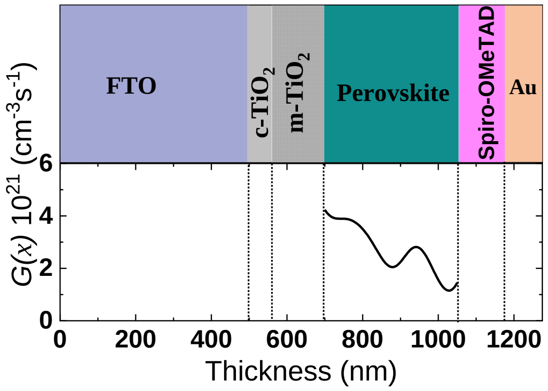

Figure S11. *(a) Spatially distributed generation rate G(x), integrated over all wavelengths under AM1.5G illumination conditions*

Integration of the above $G(x)$ distribution over perovskite thickness leads to the following value of the carrier flux under AM1.5G conditions: 0.95×10^{17} $s^{-1}cm^{-2}$. This value is similar but more precise to the above estimated absorbed photon flux N_{ph}=1.2×10^{17} $s^{-1}cm^{-2}$. The difference is due to the contribution of absorption and reflections from all layers present in our cells.

Under pulse excitation, the initial distributions of the charges are the following [S1]:

$$n(z) = n_0\, exp(-\alpha z)$$

where: $n_0 = \alpha J$, α is the absorption coefficient (in cm^{-1}), J is pump fluence (in photons/cm^2). Figure S12 presents the examples for 475 nm (α= 0.0190 nm^{-1}) and 680 nm (α = 0.0045 nm^{-1}) for 30 μJ/cm^2 fluence.

On the time scale of ps and ns these distributions further evolve due to charge diffusion, higher-order recombination, and charge extraction to contact materials [S1].

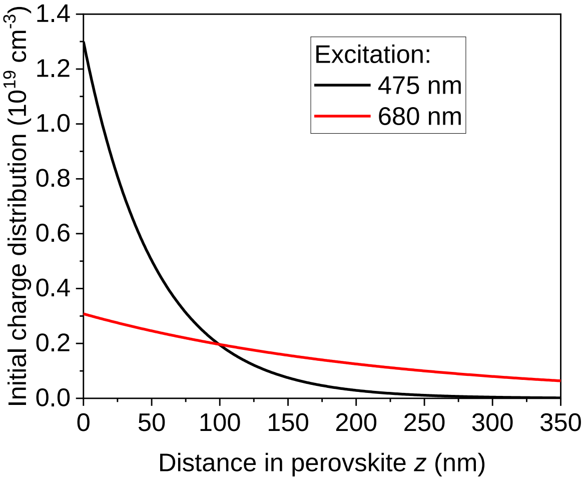

Figure S12. *Initial distribution of photogenerated charges within perovskite quickly after pulse excitation at different excitation wavelengths at 30 μJ/cm² fluence.*

If we consider the time of pulse duration (τ_{pulse}=100 fs), then the initial generation rate close to the interface is given by (for 475 nm excitation):

$$G(0) = \frac{n_o}{\tau} = 1.3 \times 10^{32}\ \ s^{-1}cm^{-3},$$

which is 11 orders of magnitude more than in the stationary conditions. However, if we consider the average generation rate, the above value has to be multiplied by the τ_{pulse}/(2 ms) ratio (repetition rate of the laser 500 Hz), which gives the generation rate:

$$G(0) = 6.5 \times 10^{21}\ \ s^{-1}cm^{-3}.$$

This value is of the same order as for continuous 1Sun illumination. Thus, depending on the time scale of the processes that we consider, the conditions at pulsed excitation can be either much stronger or similar to those at 1Sun.

Comparison of perovskite ion segregation on mesoporous TiO_2 and compact SnO_2 layers

To compare the influence of mesoporous TiO_2 on the ion segregation an alternative ETL material giving a similar number of injected electrons (photocurrent) needs to be used, therefore SnO_2 has been chosen. Standard procedure was used for SnO_2 NPs layer deposition (using commercial Alfa Aesar, 4 nm particles, from H_2O dispersion).[6] Both perovskite $(FA_{0.76}/MA_{0.19}Cs_{0.05}Pb(I_{0.81}Br_{0.19})_3)$ and spiro-OMeTAD layers were deposited during the same preparation cycle on the two substrates (TiO_2 and SnO_2). The preliminary results clearly show that the photoinduced ion segregation in mixed halide perovskite, observed when in contact with TiO_2, is significantly suppressed or even vanishes completely when the ETL material is replaced with SnO_2 (Figure S13). It should be noted that in both configurations very similar J_{sc} were observed (Table S2).

Table S2. *Parameters of the best device*:

ETL	V_{oc} [V]	*FF*	J_{sc} [mA/cm^2]	*PCE* [%]
SnO_2	1.06	0.62	15.37	10.09
$cTiO_2/mTiO_2$	1.02	0.65	15.72	10.39

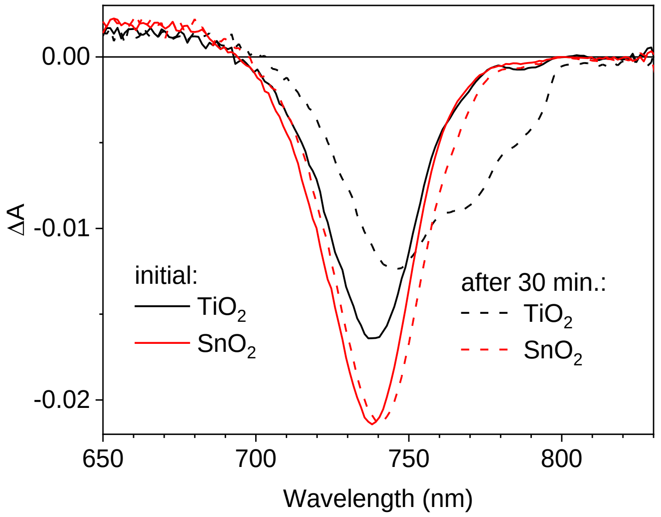

Figure S13. *Change in the TA spectra (at exemplary pump-probe delay of 8 ps) after 30 minutes of pulsed irradiation (2.5 μJ/cm²).*

The double-AG transition model – details

First, TA spectra recorded at 2 ps pump-probe delay time were globally fitted using the mono- and double-exponential functions with constant offset component for all spectra measured during the ion segregation process (each 3 min from 0 to 189 min). It was found that the mono-exponential fit ($\Delta A(E,t) = A(E)\ \exp(-t/\tau_T) + y_0(E)$) for all samples is of very good quality (r^2>0.98). The fitted time constant τ_T is shown in Figure 6a for all pump fluences, while Figure S14 presents examples of the obtained spectra of the amplitude A and of the constant offset y_0 for two selected low and high fluences. It can be noticed that the constant offset spectra have significantly different positions of the bleach peak for low and high pump fluence, which means a different final bleach spectrum of the sample when the segregation process is equilibrated. It indicates a different degree of segregation and/or iodide content in the segregated phase and will be explored below. When the double-exponential fitting was checked, an additional fast time constant of (on average) 5 min was revealed which might indicate a two-step segregation process. However, the improvement in fit quality (r^2) using the double- instead of mono-exponential global fitting was less than 0.5%, so the mono-exponential approximation used below is clearly justified.

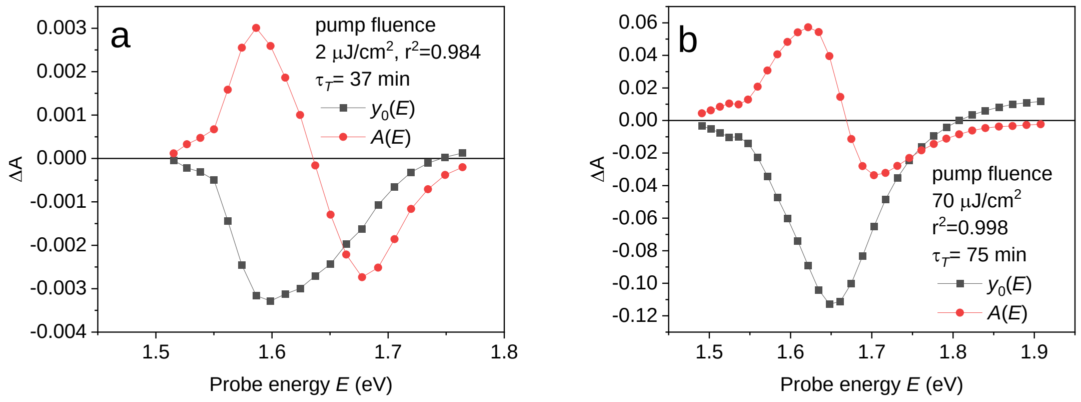

Figure S14. *Example of the pre-exponential factor and constant offset spectra obtained from mono-exponential global fit to the TA spectra evolving during ion segregation. The data were averaged each 5 points (in eV scale). The pump fluence, global fit quality (*r^2*) and fitted time constant are shown in insets.*

Then in the model, it is assumed that both phases give bleaching band signals described with an asymmetric Gaussian function (AG) and characterized by two width parameters (w_L and w_H) which are equal for both curves, i.e. the bleaching band shape is the same for both signals. However, the signal curves can have different heights (h_1 for the initial phase, and h_2 for the newly formed phase). The curves are centred at energies determined by the bandgaps of two phases (E_{c1} for the initial, mixed phase). The growing phase bandgap is related to E_{c1} by the bandgap difference parameter (ΔE_c). The AG function offset values are kept at zero to minimize the number of free parameters. As described in the manuscript, D_S stands for the degree of segregation and the transition time constant (τ_T) is fixed, based on the value obtained from direct mono-exponential global fitting to signal dynamics (described above). Eventually, a function of the following structure is fitted globally to TA spectra collected at different moments in time (parameter t):

$$\begin{aligned}\Delta A = & \left(1 - D_S\left(1 - e^{(-t/\tau_T)}\right)\right) \cdot AG_1\left(E, E_{c1}, h_1, w_L, w_H\right) \\ & + \left(D_S\left(1 - e^{(-t/\tau_T)}\right)\right) \cdot AG_2\left(E, E_{c1} - \Delta E_C, h_2, w_L, w_H,\right).\end{aligned} \quad \text{(S1)}$$

Although the function has 7 free parameters (D_S, E_{c1}, ΔE_c, h_1, h_2, w_L, w_H) all of them have justified physical meaning, and thus, overfitting or overparameterization should not take place. In fact, the model involves as few variables as possible to describe the investigated process. The assumptions that there is just one time constant and that two signals are of the same shape all simplify the phenomenon but allow us to obtain reliable results.

In the study, the global fitting involved 15 selected spectra, collected after 3, 6, 9, 13, 16, 22, 32, 41, 50, 63, 79, 95, 126, 158, and 189 minutes of irradiation. All the fittings were performed in Origin software using the Levenberg-Marquardt algorithm, with tolerance 1E-9 and derivative delta equal to 0.005. Initial values of the parameters which were used are collected in Table S3.

Table S3. *Initial parameters in the double-AG fitting.*

Parameter	D_S	τ_T *(min)*	h_1	h_2	w_L *(eV)*	w_H *(eV)*	E_{c1} *(eV)*	ΔE_C*(eV)*
Initial value	0.9	n/a	-0.1	-0.1	0.05	0.05	1.68	0.065

As it turns out, the model gives good fitting quality, with the r^2 value for all the investigated cases being higher than 0.96. What is important, the dependencies between parameters were usually below 0.9, with just 3 cases out of 63 exceeding this value and the highest one being 0.915 (for the E_{C1} parameter for 45 μJ/cm^2 fluence). This indicates that the function is not overparameterized.

Interestingly, the fitting quality measured by r^2 was the best for transitional spectra and relatively low for the initial ones, with Figure S15 showing the average r^2 for different time points. It might be caused by some fast process of different time constants taking place in the initial minutes of irradiation (as already observed above with double-exponential global fitting) – it has already been proposed that ion segregation takes place in two steps.[12]

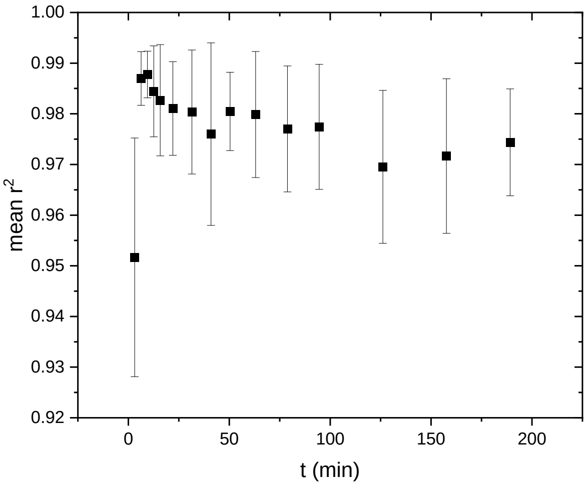

Figure S15. *Mean r^2 value by the time of the measurement.*

References:

[1] K. Pydzińska-Białek, V. Drushliak, E. Coy, K. Załęski, J. Flach, J. Idígoras, L. Contreras-Bernal, A. Hagfeldt, J. A. Anta, M. Ziółek, *ACS Appl. Mater. Interfaces* **2020**, *12*, 30399.

[2] K. Szulc, K. Pydzińska-Białek, M. Ziółek, *Materials* **2023**, *16*, 7110.

[3] A. Hagfeldt, G. Boschloo, L. Sun, L. Kloo, H. Pettersson, *Chem. Rev.* **2010**, *110*, 6595.

[4] J. Sobuś, B. Gierczyk, G. Burdziński, M. Jancelewicz, E. Polanski, A. Hagfeldt, M. Ziółek, *Chemistry – A European Journal* **2016**, *22*, 15807.

[5] N. Schopp, V. V. Brus, J. Lee, G. C. Bazan, T.-Q. Nguyen, *Advanced Energy Materials* **2021**, *11*, 2002760.

[6] H. P. Parkhomenko, M. M. Solovan, S. Sahare, A. I. Mostovyi, D. Aidarkhanov, N. Schopp, T. Kovaliuk, M. Kaikanov, A. Ng, V. V. Brus, *Advanced Functional Materials* **2024**, *34*, 2310404.

[7] D. I. Yakubovsky, A. V. Arsenin, Y. V. Stebunov, D. Y. Fedyanin, V. S. Volkov, *Opt. Express, OE* **2017**, *25*, 25574.

[8] Y. Jiang, I. Almansouri, S. Huang, T. Young, Y. Li, Y. Peng, Q. Hou, L. Spiccia, U. Bach, Y.-B. Cheng, M. A. Green, A. Ho-Baillie, *J. Mater. Chem. C* **2016**, *4*, 5679.

[9] X. Zhang, J. Qiu, X. Li, J. Zhao, L. Liu, *Appl. Opt., AO* **2020**, *59*, 2337.

[10] E. Raoult, R. Bodeux, S. Jutteau, S. Rives, A. Yaiche, D. Coutancier, J. Rousset, S. Collin, in *36th European Photovoltaic Solar Energy Conference and Exhibition*, **2019**, pp. 757–763.

[11] S. V. Zhukovsky, A. Andryieuski, O. Takayama, E. Shkondin, R. Malureanu, F. Jensen, A. V. Lavrinenko, *Phys. Rev. Lett.* **2015**, *115*, 177402.

[12] K. Suchan, J. Just, P. Beblo, C. Rehermann, A. Merdasa, R. Mainz, I. G. Scheblykin, E. Unger, *Advanced Functional Materials* **2023**, *33*, 2206047.
